# Biochar-Augmented Anaerobic Digestion System: Insights from an Interpretable Stacking Ensemble Deep Learning

**DOI:** 10.1021/acs.est.5c05051

**Published:** 2025-07-18

**Authors:** Muzammil Khan, K. C. Surendra, Sachita Baniya, Jay H. Rhymer, Samir Kumar Khanal

**Affiliations:** † Department of Molecular Biosciences and Bioengineering (MBBE), 3949 University of Hawai‘i at Ma̅noa, 1955 East-West Road, Honolulu, Hawaii 96822, United States; ‡ Department of Civil, Environmental and Construction Engineering (CECE), 3949 University of Hawai‘i at Ma̅noa, 2540 Dole Street, Honolulu, Hawaii 96822, United States; § School of Engineering and Energy, College of Science, Technology, Engineering & Mathematics, 5673 Murdoch University, 90 South Street, Murdoch, Western Australia 6150, Australia; ∥ 115513 Punahou School, 1601 Punahou St, Honolulu, Hawaii 96822, United States; ⊥ Department of Civil and Environmental Engineering, The Hong Kong University of Science and Technology, Clear Water Bay, Kowloon 999077, Hong Kong; # Affiliate faculty, Department of Environmental Engineering, Korea University Sejong Campus, Sejong-ro 2511, Sejong 2511, Korea

**Keywords:** artificial intelligence, optimization, memetic algorithm, resource recovery, waste management

## Abstract

This study presents a comprehensive approach for optimizing biochar-augmented anaerobic digestion (AD) system through an interpretable stacking ensemble deep learning model. Extensive experimental data were compiled, incorporating feedstock characteristics, operational conditions, and biochar properties, alongside stability indicators such as pH, volatile fatty acid concentrations, alkalinity, and total ammonia nitrogen levels. The proposed model integrates different configurations of convolutional neural networks and long short-term memory networks within a stacking ensemble framework, effectively capturing complex interdependencies within the AD process and improving methane yield predictions. Optimized through advanced hyperparameter tuning, the model achieved high internal predictive accuracy (mean *R* ^2^ of 0.91–0.94 and root-mean-square error of 60.85 mL CH_4_/g volatile solids) and demonstrated strong generalization with an *R* ^2^ of 0.68 on external independent lab-scale datasets, outperforming all individual models. Post-hoc interpretability analysis using permutation importance and Shapley Additive Explanations (SHAP) identified critical factors influencing methane production and stability indicators. A model-based global optimization framework was implemented to tailor optimal operational conditions for real-world scenarios, ensuring high methane yield while maintaining process stability. Additionally, a user-friendly graphical interface was developed to facilitate the practical implementation of the predictive model. This work provides a robust framework for optimizing the AD process with biochar augmentation, enhancing resource recovery and waste management.

## Graphical abstract

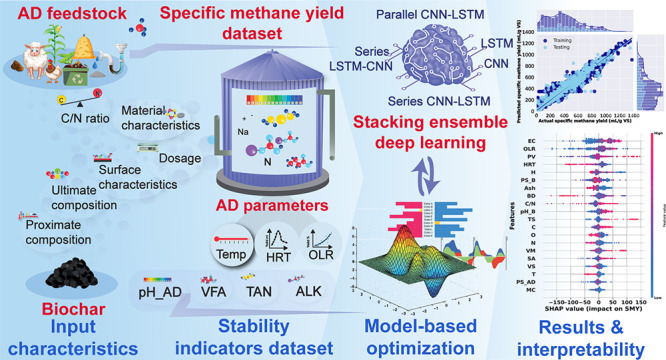

## 1 Introduction

Anaerobic digestion (AD) is a well-established and widely adopted technology for treating diverse organic wastes, enabling simultaneous resource recovery, including renewable energy and organic fertilizer. The performance of AD systems is governed by various factors, such as feedstock types, environmental conditions, and digester operational parameters. Consequently, studies have explored different strategies to improve AD system performance, particularly in terms of digester stability and methane yield. These strategies include co-digestion,^1^ feedstock pretreatment,^2^ nutrient supplementation,^3^ nanobubble technology,^4^ microaeration,^5^ and biochar augmentation.^6^

Among these strategies, biochar augmentation has emerged as a promising approach to enhancing AD performance.^7^ Biochar, a carbon-rich material produced through biomass pyrolysis, exhibits unique properties, such as high surface area, porosity, stability, and diverse functional groups, all of which contribute to improved AD system performance.^8,9^ Biochar enhances process stability by promoting microbial growth through its immobilization effect,^10^ facilitating the direct interspecies electron transfer (DIET) between syntrophic bacteria and methanogens,^11^ increasing the digester’s buffer capacity,^12^ and adsorbing inhibitory compounds,^13^ ultimately leading to increased methane yield.

Both feedstock types and production conditions govern the key characteristics of biochar, including surface properties,^14^ electrochemical properties,^15^ proximate composition, and ultimate composition.^13^ Biochar’s unique properties influence AD system performance in different ways.^16,17^ For example, biochar’s high surface area and porosity promote microbial growth,^18^ while its high electrical conductivity enhances electron transfer and carbon dioxide reduction, ultimately improving methane yield.^19^ Thus, understanding the fundamental physicochemical properties of biochar that influence AD system performance is crucial.^13,14^ While experimental studies provide valuable insights into biochar’s specific effects on AD system performance, they are often time-consuming and costly. Mechanistic models like Anaerobic Digestion Model No. 1 (ADM1) and modified Hill’s model offer alternatives but are complex, require extensive prior knowledge, and are limited in scope as they lack the capability to incorporate biochar-specific parameters.^20,21^

Artificial intelligence (AI) has emerged as a promising modeling tool for predicting and optimizing the performance of biological processes, including AD.^22^ These models are ideally suited to capture complex, nonlinear behaviors inherent in biological systems like AD process that are difficult to model mechanistically. Various machine learning (ML) algorithms have been explored for performance prediction of biochar-augmented AD systems, including neural networks,^23–26^ tree-based models,^27^ and automated ML models.^28^ However, these studies often exhibit methodological and scope-related limitations that constrain broader applicability. Many studies focus on a single or limited range of substrates (e.g., sewage sludge, cheese whey, manure),^23–26^ rely on batch systems and use small datasets (typically 14–51 data points),^23–26,28^ limiting the generalizability of the results. The input features are often narrow, such as total solids, biochar dosage, or carbon-to-nitrogen (C/N) ratio, while key biochar physicochemical properties (e.g., proximate composition, pH, electrical conductivity, surface area) and critical AD operation parameters (e.g., organic loading rate, particle size, hydraulic retention time, solids retention time) are frequently overlooked. Most of these models target methane or biogas yield prediction, with limited attention to system stability or biochar-AD parameter interactions. Even advanced ML frameworks, including AutoML, have not fully addressed substrate and biochar diversity or the complexity of their interactions within the AD process.^28^ The reliance on batch systems further limits the relevance of these findings to continuous or semicontinuous operations. Li et al. attempted to address some of these issues by comparing various ML algorithms and focusing on general AD system parameters.^29^ However, except for biochar dosage, the study did not consider other critical biochar characteristics and their potential interactions with AD system parameters. To overcome these limitations, a comprehensive approach that incorporates detailed biochar characteristics and key AD system parameters is needed. A detailed comparison of these studies, including their inputs, modeling techniques, data sizes, and outputs, is provided in Table S5 in Supporting Information.

Advanced deep learning (DL) algorithms, such as convolutional neural networks (CNNs) and long short-term memory networks (LSTMs), are particularly suited for modeling the performance of the biochar-augmented AD system due to their robust capability in capturing the complex interdependencies between parameters.^30,31^ Despite their potential, the impact of different architectural configurations on the effectiveness of these models remains largely underexplored. Stacking ensemble DL, a method that integrates multiple models, has shown significant promise in enhancing predictive accuracy and robustness.^32,33^ Integrating CNNs and LSTMs within a stacking ensemble framework could more effectively capture complex interactions in biochar-augmented AD systems and methane yield dynamics, thereby improving overall model performance.

This study addresses these challenges by providing an in-depth analysis of advanced DL algorithms for predicting and optimizing the performance of biochar-augmented semicontinuous AD systems. A novel stacking ensemble DL model is proposed, incorporating various base models, including standalone architectures (CNN and LSTM), sequential architectures (series CNN-LSTM and series LSTM-CNN), and parallel architecture (parallel CNN-LSTM), to predict methane yield. Furthermore, data on AD system stability indicators were compiled to enable a comprehensive understanding of biochar-augmented AD system performance. An interpretability analysis was employed to elucidate the contribution of each parameter to methane yield. To enhance the model’s practicality and gain deeper insights, a model-based global optimization approach was implemented to tailor optimal environmental conditions and operational parameters for the biochar-augmented AD systems.

## 2 Materials and Methods

### 2.1 Data Collection from Experimental Studies

Data from experimental studies on biochar-augmented semicontinuous AD systems, published between 2018 and 2024, were collected from articles available on the Web of Science and Google Scholar. Numerical data were extracted from manuscript descriptions and tables, while data from figures were collected using Web Plot Digitizer software (https://apps.automeris.io/wpd/). Data were collected on key AD feedstock characteristics ((total solids (TS), volatile solids (VS), C/N ratio)), AD system operational parameters ((organic loading rate (OLR), hydraulic retention time (HRT), temperature, particle size)), and biochar properties (electrical conductivity, specific surface area, pore volume, biochar pH, particle size, proximate composition (volatile matter, fixed carbon, moisture content, ash content) and ultimate composition (carbon, hydrogen, nitrogen, and oxygen contents)). The primary output parameter analyzed was the specific methane yield (SMY). To gain deeper insights into the biochar-augmented AD systems, additional data were collected on AD system stability indicators, including digester pH, volatile fatty acids (VFAs) concentration, alkalinity (ALK), and total ammonia nitrogen (TAN) content. A detailed description of the dataset, including search criteria and keywords used for article selection, is provided in Text S1 in the Supporting Information.

### 2.2 Dataset Organization and Preprocessing

To facilitate comprehensive analysis and modeling, the collected data were organized into two distinct datasets: the SMY-dataset and the stability indicator-dataset. The SMY-dataset, containing approximately 5,000 experimental data points, was designed to predict the performance of biochar-augmented AD systems, with SMY as the primary output parameter. It included all the previously mentioned input parameters, such as feedstock characteristics, digester operating parameters, environmental conditions, and biochar properties. The stability indicator-dataset, comprising about 2000 experimental data points, was specifically created to model the stability of biochar-augmented AD system. While using the same input parameters as the SMY-dataset, the stability indicator-dataset focused on different output parameters, such as digester pH, VFA concentration, ALK, and TAN content. The workflow diagram summarizing the methodology is illustrated in Figure 1. Prior to developing DL models, data preprocessing was implemented to ensure data quality and consistency. MissForest method was employed for data imputation.^34^ A preliminary analysis was conducted to explore the variables in biochar-augmented AD systems, including data visualization using violin plots (Figure S1a) and a descriptive summary (Table S1). To further examine relationships between inputs and outputs for developing DL predictive models, Pearson’s correlation coefficient (PCC) and Spearman’s rank coefficient (SRC) were used to assess linear and monotonic correlations between parameters (Figure S1a). Detailed information on data preprocessing (Text S1) and PCC and SRC correlation analyses (Texts S2 and S3) is provided in the Supporting Information.

**Figure 1:**
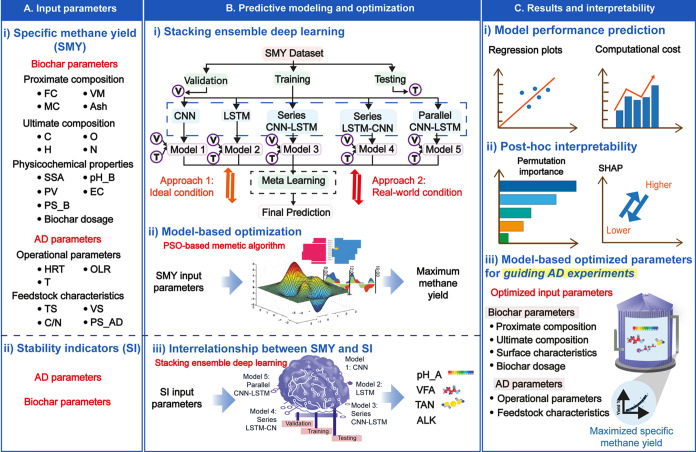
Schematic framework of proposed methodology for modeling and optimization of a biochar-augmented AD system. (AD: Anaerobic digestion; OLR: Organic loading rate; HRT: Hydraulic retention time; T: Temperature; C/N: Carbon/nitrogen ratio; VS: Volatile solids; TS: Total solids; PS_AD: Particle size of AD feedstock; BD: Biochar dosage; SSA: Specific surface area; PV: Pore volume; pH_B: pH of biochar; PS_B: Particle size of biochar; VM: Volatile matter; FC: Fixed carbon; MC: Moisture content; C: Carbon; H: Hydrogen; N: Nitrogen; O: Oxygen; pH_AD: Digester pH; VFA: Volatile fatty acid; ALK: Alkalinity; and TAN: Total ammonia nitrogen).

### 2.3 Model Development and Evaluation

For base model development, 21 variables encompassing AD feedstock characteristics (TS, VS, C/N ratio, and feedstock particle size), digester operating parameters (OLR, HRT, temperature, and biochar dosage), and biochar properties (electrical conductivity, specific surface area, pore volume, biochar pH, biochar particle size, volatile matter, fixed carbon, moisture, ash, and carbon, hydrogen, nitrogen and oxygen contents) were used as inputs, with SMY as the target parameter. The SMY-dataset was divided into 80% training and 20% testing sets, and this train-test split was repeated five times with different random seeds (repeated holdout validation) to evaluate model performance and stability.^35^ Additionally, repeated five-fold cross-validation (five repeats) was performed as a supplementary check of robustness across data partitions. To explore the full potential of advanced DL models, five different models, including standalone architectures (CNN and LSTM), sequential architectures (series CNN-LSTM and series LSTM-CNN), and parallel architecture (parallel CNN-LSTM), were trained as base models. Detailed description of these architectures is provided in Text S4 in the Supporting Information. Hyperparameter optimization for each model was conducted using the Optuna framework to ensure optimal performance (Text S5).^36^ Following hyperparameter optimization, the model performance was tested on unseen testing data using root-mean-square error (RMSE) and coefficient of determination (*R* ^2^) as the performance evaluation metrics (Text S6). Finally, a stacking ensemble DL model was developed by combining the outputs of all base models (CNN, LSTM, series CNN-LSTM, series LSTM-CNN, and parallel CNN-LSTM). A meta-model was then trained on these base model outputs to generate the final predictions. Various ML algorithms were tested as potential meta-models, and the best-performing model was selected (Figure S10). For the proposed stacking ensemble DL model, data splitting, hyperparameter tuning, and model evaluation followed the same methods as the base models to ensure consistency.

### 2.4 Post-hoc Model Interpretability and Deployment

To enhance the interpretability of the stacking ensemble DL model, the influence of input parameters on SMY prediction was analyzed using permutation importance and Shapley Additive Explanations (SHAP) techniques. To further explore the feature-target dynamics, partial dependence plots (PDPs) were generated, including both one-way and two-way PDPs, to illustrate how changes in feature values affect methane yield predictions.^27,34^ SHAP analyses were conducted using the SHAP package in Python, while PDPs were created using the pdpbox package. The details of these post-hoc model interpretability methods are provided in Text S7 in the Supporting Information. Additionally, a separate DL model was developed using the stability indicator-dataset to gain insights into relationships between input parameters and process stability indicators within a biochar-augmented AD system. This complementary model not only provides insights into the factors influencing process stability but also enriches the understanding of feature importance in SMY predictions, contributing to a comprehensive understanding of overall process dynamics. Additionally, an easy-to-use graphical user interface (GUI) was developed to facilitate the practical implementation of the predictive model (Figure S13). This tool enables researchers and operators to rapidly test various operational scenarios and biochar modifications without requiring expertise in DL or programming, thereby bridging the gap between advanced modeling techniques and practical application in biochar-augmented AD systems.

### 2.5 Model-Based Optimization Framework

To enhance the practicality and effectiveness of the proposed DL model, a model-based global optimization framework aimed at maximizing SMY was implemented. Specifically, a particle swarm optimization (PSO)-based memetic algorithm integrated with the stacking ensemble DL model was employed to efficiently explore the high-dimensional parameter space and identify optimal AD system operational conditions and biochar characteristics. While PSO is a widely used global optimization technique, it is known to be susceptible to premature convergence and local optima entrapment in complex, continuous parameter spaces. To address these limitations, a hybrid memetic strategy was adopted that combines PSO’s global search capability with gradient descent-based local refinement.^37^ This integration enables the algorithm to first identify promising regions globally, and then fine-tune solutions locally, significantly enhancing convergence robustness and solution quality.^37^ PSO-based memetic approach was selected due to its suitability for continuous, high-dimensional AD system optimization tasks, offering a robust balance between global exploration and local exploitation. While other metaheuristic algorithms, such as genetic algorithms (GA) or simulated annealing (SA), could be employed, PSO-memetic was chosen for its computational efficiency and proven effectiveness in similar bioprocess optimization applications.^22^ The details about the PSO-based memetic algorithm are provided in Text S8 in the Supporting Information.

Two distinct optimization approaches were explored. The first approach optimized the entire set of the above-mentioned 21 variables, encompassing feedstock characteristics, digester operating conditions, and biochar properties, using a DL model trained on all relevant inputs. This comprehensive optimization was designed to establish near-optimal conditions for maximizing SMY, ensuring ideal digester performance. The second approach addressed the practical limitations often encountered in real-world applications, where feedstock composition cannot always be adjusted. To bridge the gap between theoretical optimization and real-world application, this method focuses on optimizing controllable operational parameters (e.g., OLR, HRT, temperature, and biochar dosage) while using fixed AD feedstock and biochar properties representative of typical practical conditions. Food waste was selected as the AD feedstock to reflect a critical and underutilized organic waste stream with substantial potential for renewable energy recovery while managing the waste. In the US, food waste is the largest material sent to landfills and incinerators, comprising about 24% of landfilled municipal solid waste and contributing significantly to landfill methane emissions.^38^ Its diversion to AD system aligns with national and state-level policy efforts to reduce organic waste disposal and mitigate methane emissions.^39^ Furthermore, the favorable characteristics of food waste, including a high volatile solids content, suitable C/N ratio, and rapid biodegradability, make it particularly suitable feedstock for biochar-augmented AD system.^40,41^ Our literature review confirmed this relevance, with food waste representing the most frequently studied substrate in recent biochar-augmented AD studies (Figure S18 in the Supporting Information). The food waste used in this study was collected over the course of a week from the cafeteria of University of Hawai‘i at Ma̅noa, homogenized, subsampled, blended for consistency, and characterized as described previously.^42^ Sewage sludge-derived biochar was selected as the additive due to its common application in AD studies. Data on its characteristics were obtained from published work,^43^ which highlights its potential in enhancing process stability and methane production during the AD of food waste.

## 3 Results and Discussion

### 3.1 Model Development for Methane Yield Prediction

#### 3.1.1 Base DL-Models

The SMY-dataset was used to develop five base DL models, including CNN, LSTM, series CNN-LSTM, series LSTM-CNN, and parallel CNN-LSTM architectures. Optimized hyperparameters for these models, determined using the Optuna framework, are presented in Figure 2d. The performance of these models for predicting SMY varied, with testing *R* ^2^ values ranging from 0.80 to 0.84 and RMSE values between 98 and 109 mL CH_4_/g VS. Regression plots illustrating the relationship between predicted and actual SMY values for all base models are shown in Figure S12 in the Supporting Information. Among these models, the CNN demonstrated superior performance with the highest testing *R* ^2^ of 0.84 and the lowest RMSE of 99 mL CH_4_/g VS. The strong predictive performance suggests its effectiveness in capturing complex feature interdependencies among variables. Its computational efficiency (run time of 19 min) makes it particularly suitable for rapid process optimization scenarios, where quick predictions of SMY based on varying biochar properties and AD feedstock parameters are needed (Figure 2d). In contrast, the LSTM model achieved predictive performance nearly identical to that of the CNN model (*R* ^2^ = 0.84; RMSE = 98 mL CH_4_/g VS) but required substantially longer computational time (156 min). This indicates that the added complexity of the LSTM architecture did not provide a meaningful additional benefit in capturing feature interdependencies for this dataset.

**Figure 2:**
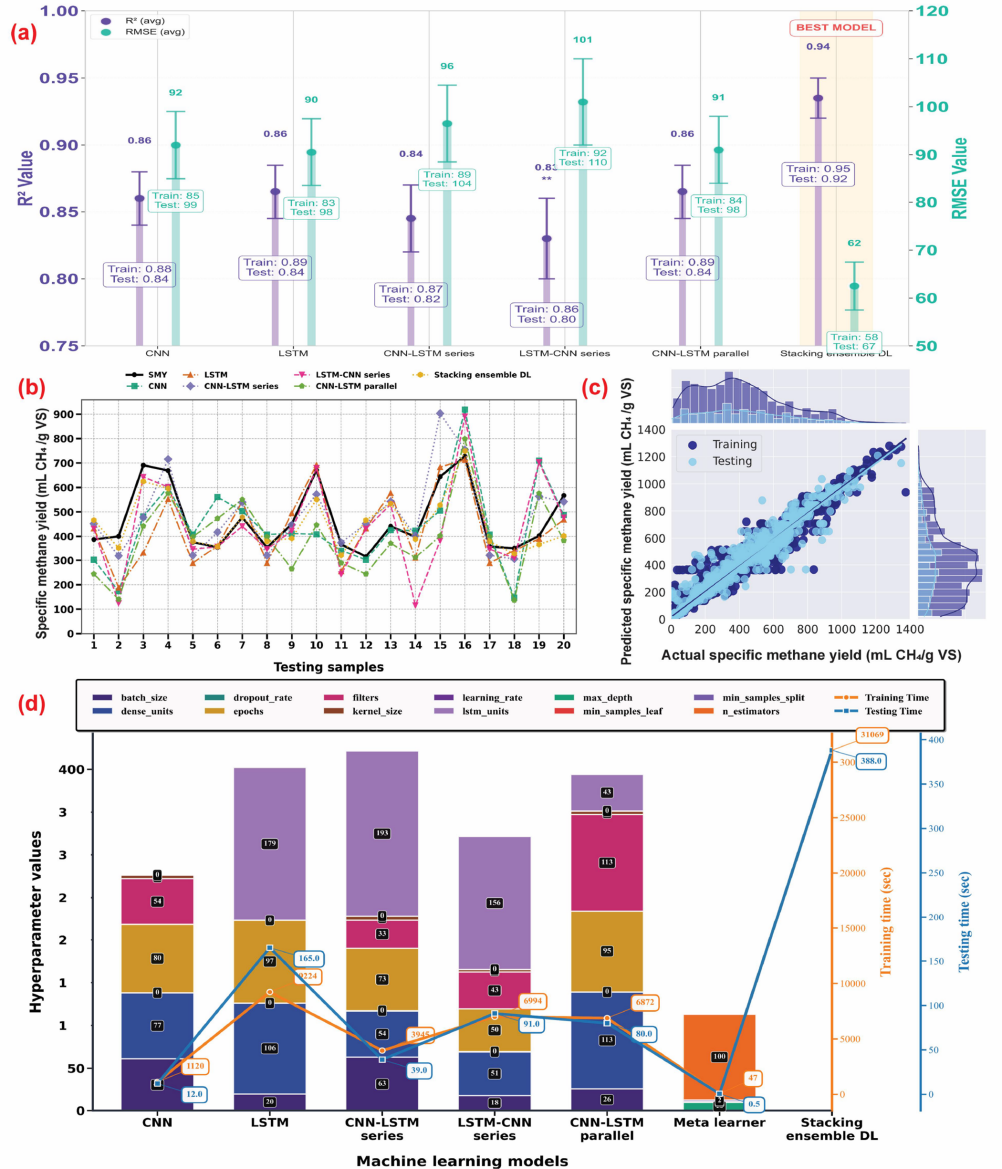
Performance evaluation of stacking ensemble deep learning (DL) model. (a) Comparison of predictive performance of various DL models. (b) Variations of specific methane yield (SMY) predicted by stacking ensemble DL model over various experimental data points. (c) Regression plot of the best model (stacking ensemble DL). (d) Optimized hyperparameters and computational cost of base and stacking ensemble DL models. (CNN: Convolutional neural network; LSTM: Long short-term memory; *R* ^2^: Coefficient of determination; RMSE: Root mean square error).

The hybrid models, particularly the CNN-LSTM parallel configuration, were developed to combine the strengths of both architectures, aiming to better capture complex feature interdependencies and improve predictive accuracy. The CNN-LSTM parallel model achieved predictive performance comparable to the individual CNN and LSTM models with a mean *R* ^2^ of 0.84 while requiring a moderate computational time of 116 min. However, the series configurations showed slightly lower performance (series CNN-LSTM with *R* ^2^ = 0.82 and series LSTM-CNN *R* ^2^ = 0.80), indicating that these models may struggle to fully integrate the complex interactions among process parameters in biochar-augmented AD systems.

These findings suggest that in a biochar-augmented AD system, the interplay between the process parameters is more complex than initially anticipated. Addressing this complexity may require a more nuanced modeling approach, such as the stacking ensemble model, which leverages multiple base models to achieve superior prediction accuracy. By capitalizing on the synergistic strengths of CNN and hybrid architectures, the stacking ensemble approach creates a comprehensive representation of process dynamics in biochar-augmented AD systems, significantly improving performance prediction and methane yield optimization. Further details on model development and evaluation are provided in Figures S2–S12 and Table S3 in the Supporting Information.

#### 3.1.2 Stacking Ensemble DL-Model

A stacking ensemble architecture, combining the aforementioned base models, was employed to improve the overall model performance for predicting and optimizing SMY in biochar-augmented AD systems. The outputs of the base models were combined and processed by meta-learner models, which were specifically trained to predict SMY. Among 38 tested meta-learners, extra trees regression outperformed the others, yielding an improved stacking ensemble DL model with the highest *R* ^2^ and the lowest RMSE (Figure S10). The stacking ensemble DL model demonstrated superior performance in predicting SMY, with a mean *R* ^2^ of 0.94 and a mean RMSE of 61 mL CH_4_/g VS (Figure 2a). This represented a substantial improvement over base models, indicating its ability to capture intricate relationships among different parameters within biochar-augmented AD systems that individual models may overlook. The significantly lower RMSE (38% reduction in prediction error compared to the next-best model) underscored the stacking ensemble’s precision in estimating SMY. Moreover, its predictive performance (*R* ^2^ = 0.94) surpassed results from recent studies (*R* ^2^ = 0.82–0.84),^29,34^ emphasizing the model’s effectiveness in this domain. However, the increased computational time (model training) of 476 min of the stacking ensemble presents a trade-off. This trade-off between predictive performance and computational efficiency highlights the need for careful consideration in practical applications, especially in scenarios requiring real-time or rapid prediction for process optimization and control. Furthermore, the repeated five-fold cross-validation produced comparable results (mean *R* ^2^ = 0.91), supporting the reliability of the model’s predictive performance (Figure S16). While detailed comparisons of computational time could not be performed, as prior studies generally did not report this metric, a comprehensive comparison of inputs, outputs, and model accuracy is provided in Table S5 in Supporting Information to contextualize results of this study within the literature.

### 3.2 Identifying and Ranking Key Parameters Influencing Methane Yield

The interpretability analysis revealed intricate interactions between biochar properties and AD system parameters that significantly influenced SMY in biochar-augmented AD systems. Permutation importance analysis initially identified OLR as the most critical factor (importance score: 0.1796), followed by biochar pore volume (0.1338) and its electrical conductivity (0.1153) (Figure 3a). However, the subsequent SHAP analysis provided a more nuanced perspective, where electrical conductivity emerged as the most influential parameter, followed by OLR, biochar pore volume, feedstock’s C/N ratio, and biochar dosage (Figure 3b). This shift in ranking between the two methods highlights that SHAP analysis can better capture the complex, nonlinear relationships between biochar’s conductive properties and enhanced syntrophic interactions between microorganisms.^15^ To further explain how these key factors affect SMY across their value ranges, a SHAP summary plot analysis was conducted.

**Figure 3:**
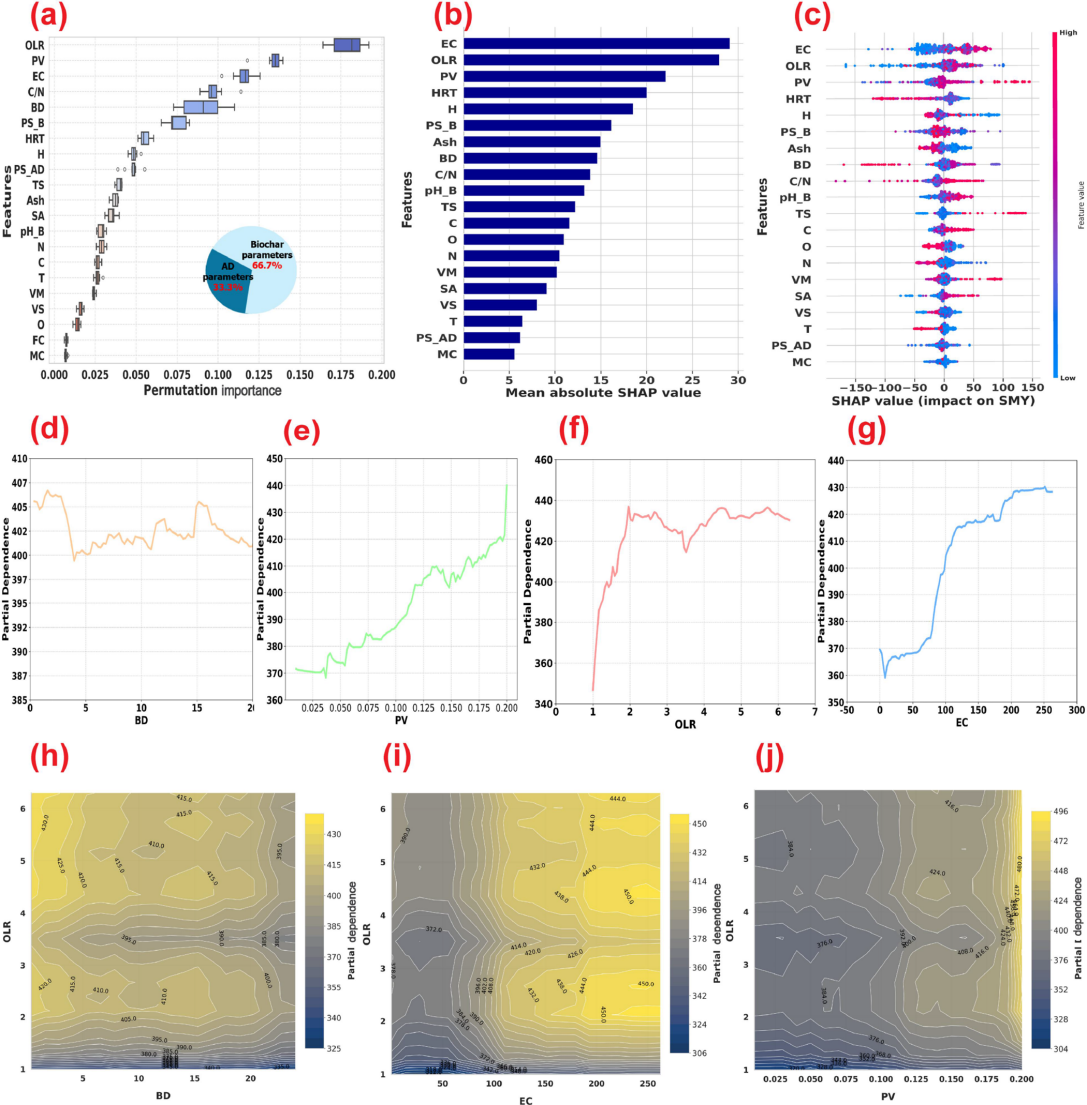
Interpretability analysis of the proposed stacking ensemble DL model. (a) Permutation importance. (b) Mean absolute SHAP values. (c) SHAP summary plot. (d) One-way partial dependence plots (PDPs) of organic loading rate OLR. (e) One-way PDP of electrical conductivity (EC). (f) One-way PDP pore volume (PV). (g) One-way PDP biochar dosage (BD). (h) Two-way PDP of OLR versus BD. (i) Two-way PDP of OLR versus EC. (j) Two-way PDP of OLR versus PV. (AD: Anaerobic digestion; OLR: Organic loading rate; HRT: Hydraulic retention time; T: Temperature; C/N: Carbon/nitrogen ratio of AD feedstock; VS: Volatile solids content of AD feedstock; TS: Total solids content of AD feedstock; PS_AD: Particle size of AD feedstock; BD: Biochar dosage; SSA: Specific surface area of biochar; PV: Pore volume of biochar; pH_B: pH of biochar; PS_B: Particle size of biochar; VM: Volatile matter; FC: Fixed carbon; MC: Moisture content; C: Carbon content; H: Hydrogen; N: Nitrogen; O: Oxygen; pH_AD: Digester pH; VFA: Volatile fatty acid; ALK: Alkalinity; and TAN: Total ammonia nitrogen).

The SHAP summary plot revealed the electrical conductivity’s complex, nonlinear relationship with SMY (Figure 3c). While higher electrical conductivity values generally exhibited a positive impact, the broad distribution of SHAP values indicated their context-dependency and potential interaction with other parameters. This nonlinearity emerged because methane production in AD is more strongly influenced by biochar’s electron-donating capacity (EDC), governed by its redox-active functional groups, rather than by its bulk electrical conductivity.^44^ While electrical conductivity can aid in DIET,^45^ biochar with lower electrical conductivity but higher redox activity has shown superior performance in enhancing methane production.^15^

The PDP for OLR showed that SMY initially increases with increasing OLR, which reflects improved substrate availability and microbial activity (Figure 3f). However, beyond a threshold, SMY stabilized rather than declining, suggesting that biochar amendment provides buffering capacity that prevents the process inhibition typically observed at high OLRs.^46^ This phenomenon contrasts with the performance of conventional AD systems without biochar amendment, where high OLR often leads to VFAs accumulation, resulting in reactor acidification, and ultimately, failure.^47^ In biochar-augmented AD systems, biochar contributes to this resilience by buffering pH, adsorbing VFAs, and providing a large surface area for microbial attachment and growth, thereby improving process stability.^46,48^ For instance, biochar supplementation has been associated with a 12% increase in methane yield at OLRs of 3.13–6.25 g VS/L·d and improved tolerance to TAN levels above 2450 mg/L, which often represent the inhibition threshold for digesters operated under high OLRs.^46,48^ Additionally, biochar amendment has also been linked to microbial community shifts that influence methanogenic populations, with biochar supplementation promoting the dominance of Methanosarcina compared to control digesters where strictly aceticlastic Methanosaeta was dominant.^48^ This shift is significant, as Methanosarcina utilizes various substrates, including acetate, methanol, methylamine, hydrogen, and carbon dioxide, enabling multiple methanogenesis pathways and enhancing methane production via metabolic versatility and DIET-based syntrophic interactions. This microbial community change improves digester performance even at a high OLR (6.25 g VS/L·d), demonstrating biochar’s ability to enhance process stability under stressed conditions.^48^

Furthermore, biochar potentially facilitates DIET and accelerates propionic acid degradation, thereby preventing the inhibition of methanogenesis. However, the plateau in SMY at high OLRs suggests that while biochar amendment extends the OLR tolerance threshold, it does not indefinitely enhance methane yield. Instead, biochar amendment primarily stabilizes reactor performance, reducing the risk of system failure rather than substantially increasing methane yield beyond a certain point. These findings highlight the importance of optimizing OLR and suggest that biochar amendment should be considered as one of several complementary strategies for improving AD system stability under high OLR conditions.

Biochar pore volume demonstrated a positive correlation with SMY, with higher values linked to elevated SHAP values, particularly in the upper range (Figure 3g). This suggests a potential threshold effect, where increased biochar pore volume significantly enhances microbial colonization and substrate utilization. Studies have shown that biochar with higher pore volume provides more surface area for microbial attachment, improves mass transfer, and enhances substrate accessibility, ultimately boosting methane yield.^49,50^

The C/N ratio of AD feedstock showed a complex relationship with SMY, with both low and high values enhancing methane yield depending on factors such as substrate composition and biochar properties.^51^ At low C/N ratios, where ammonia inhibition is common, biochar augmentation can increase SMY by adsorbing excess ammonia and stabilizing the AD process.^51^ Conversely, at high C/N ratios, which may limit microbial growth, biochar augmentation can boost methane yield by enhancing microbial growth and activity.^52^ Thus, the optimal C/N ratio in biochar-augmented AD systems varies based on these interactions. Furthermore, biochar dosage generally showed a positive correlation with SMY, with higher dosages often yielding positive SHAP values. However, the value spread suggested that optimal biochar dosage depends on interactions with other parameters, including TS content of the AD substrate, biochar type/feedstock source, the C/N ratio of AD substrate, and HRT.^53,54^

The PDP for electrical conductivity revealed a nonlinear relationship with SMY (Figure 3f). As electrical conductivity increased from 0 to about 2 mS/cm, there was a significant increase in SMY, indicating a significant enhancement in methane production. Beyond 2 mS/cm, the rate of increase slowed down, continuing up to about 6 mS/cm before plateauing, suggesting an optimal electrical conductivity range of 2–6 mS/cm. This can be attributed to enhanced electron transfer, improved nutrient availability, and the formation of biofilm facilitated by higher electrical conductivity levels.^15,55^ For OLR, the PDP showed a strong positive correlation with SMY up to about 3 g VS/L·d, indicating efficient organic matter conversion within this range. However, at higher OLRs, the SMY decreased, which was likely due to VFAs accumulation leading to microbial inhibition.^47,56^ The PDP for biochar pore volume demonstrated a positive correlation with SMY, with a steep increase up to approximately 0.4 cm^3^/g, followed by a more gradual rise. This underscored the critical role of biochar’s porous structure in enhancing AD system performance through improved microbial colonization and adsorption of inhibitory compounds. Highly porous biochar further boosts performance by providing protective microhabitats and increased adsorption capacity.^49^ The PDP for C/N ratio revealed a complex, nonmonotonic relationship with SMY. SMY initially increased when the C/N ratio increased from 10 to about 25, followed by a slight decrease and another increase at higher ratios (>28). This suggests an optimal C/N range of around 20–25, balancing carbon and nitrogen availability for microbial growth. The complex relationship indicated the need for careful nutrient balance in biochar-augmented AD systems.^57^ Lastly, the PDP for biochar dosage showed a rapid increase in SMY as dosage increased to about 1 g/L, followed by a more gradual increase up to 5 g/L (Figure 3d). While higher biochar dosage generally improves SMY, excessive biochar dosage can inhibit methane production due to intermediates accumulation, nonselective gas adsorption, and metal toxicity.^53,58^ High biochar dosages often lead to reduced methane yield due to microbial inhibition and decreased system stability.^52,59^

The two-way PDP for electrical conductivity vs OLR revealed a complex interplay between these critical parameters (Figure 3i). The highest SMY values (∼450 mL CH_4_/g VS) were observed at high biochar electrical conductivity values (>200 μS/cm) combined with high OLRs (4–6 g VS/L·d), indicating that these conditions are optimal for methane production. In contrast, low electrical conductivity values (<100 μS/cm) and low OLRs (<2 g VS/L·d) corresponded to the lowest SMY values (∼306 to 324 mL CH_4_/g VS). These findings imply that increasing both electrical conductivity and OLR promotes methanogenesis, which was likely due to enhanced microbial activity and improved electron transfer facilitated by biochar under high substrate availability and electrical conductivity conditions.^15,48^ This trend, however, contradicts the findings of previous studies,^34^ which reported the most favorable methane yields at low electrical conductivity levels (∼60 μS/cm).^15^ Those studies attributed the higher methane yield at low electrical conductivity to the abundance of redox-active functional groups on biochar surfaces, such as hydroquinone, facilitating functional group-mediated electron transfer.^44^ However, those results were based on one-way analyses, where electrical conductivity was studied in isolation, and did not account for interactions with other critical operational parameters such as OLR. It is possible that under certain conditions, low electrical conductivity plays a more dominant role in driving methanogenesis, particularly when other parameters are held constant. Conversely, the two-way PDP suggested that at high OLRs, the combined effects of increased substrate availability and electrical conductivity could enhance methane production, potentially by complementing redox-active functional groups with DIET.^15,44^ These differences highlight the complexity of biochar’s role in the AD system and suggest that both the direct and interactive effects of electrical conductivity must be considered to fully understand its impact on methanogenesis.

The two-way PDP for biochar pore volume and OLR showed that the highest SMY values (∼496 mL CH_4_/g VS) were concentrated in the upper right corner, corresponding to high biochar pore volume (≥0.18 cm^3^/g) and moderate to high OLR values (>4 g VS/L·d) (Figure 3j). In contrast, at lower pore volume values (<0.05 cm^3^/g), SMY decreased noticeably, especially at low OLRs. For instance, at an OLR of 1 g VS/L·d, SMY dropped from ∼360 to ∼304 mL CH_4_/g VS as pore volume decreased. These observations suggest that biochar with higher pore volumes could help mitigate performance declines by enhancing substrate mass transfer and providing suitable microenvironments for microbial colonization.^49^ Additionally, high pore volume may buffer the negative effects of high OLRs, as SMY remained relatively stable (∼450 to 496 mL CH_4_/g VS) when pore volume was ≥0.15 cm^3^/g, even at high OLRs (>4 g VS/L·d). Studies have reported such stabilization effects specifically in systems utilizing biochar with well-developed pore networks, which provide enhanced mass transfer and microbial habitat advantages.^49^

The two-way PDP plot between biochar dosage and OLR showed that the highest SMY values (∼430 mL CH_4_/g VS) were achieved at moderate biochar dosages (5–10 g/L) and moderate OLRs (2–4 g VS/L·d) **(** Figure 3h). In contrast, excessive biochar dosages (>15 g/L) were associated with substantial declines in SMY, particularly at low OLRs (<3 g VS/L·d). For instance, at an OLR of 1 g VS/L·d, SMY dropped from ∼395 to ∼325 mL CH_4_/g VS as biochar dosage increased from ∼5 to ∼20 g/L. At moderate biochar dosages (∼10 g/L), increasing OLR to 6 g VS/L·d stabilized SMY at ∼410 mL CH_4_/g VS, while higher biochar dosages did not improve methane yield, but rather exacerbated process inhibition. These results suggest that moderate biochar dosages (5–10 g/L) are optimal for enhancing methane production in biochar-augmented AD systems, while excessive dosages may hinder performance. This trend aligns with previous findings demonstrating biochar’s stimulatory effects on biogas production at low OLRs (3–4 g VS/L·d), which diminish at high OLRs (up to 7 g VS/L·d).^60^ The decline in performance at high OLRs is likely due to the increase in VFA and ammonia concentrations, a typical phenomenon at high OLRs, counteracting the positive effects of biochar.^60^

### 3.3 Interrelationships between AD System Stability Indicators and Methane Production

Biochar-augmented AD represents a complex ecosystem where process stability and methane production are intricately linked (Figure 4). The SHAP analysis identified the top ten features influencing biochar-augmented AD system performance, with OLR, operating temperature, and C/N ratio being the most critical (Figure 4b). Biochar properties, including specific surface area, particle size, and hydrogen and fixed carbon contents, emerged as key factors affecting stability indicators.

**Figure 4:**
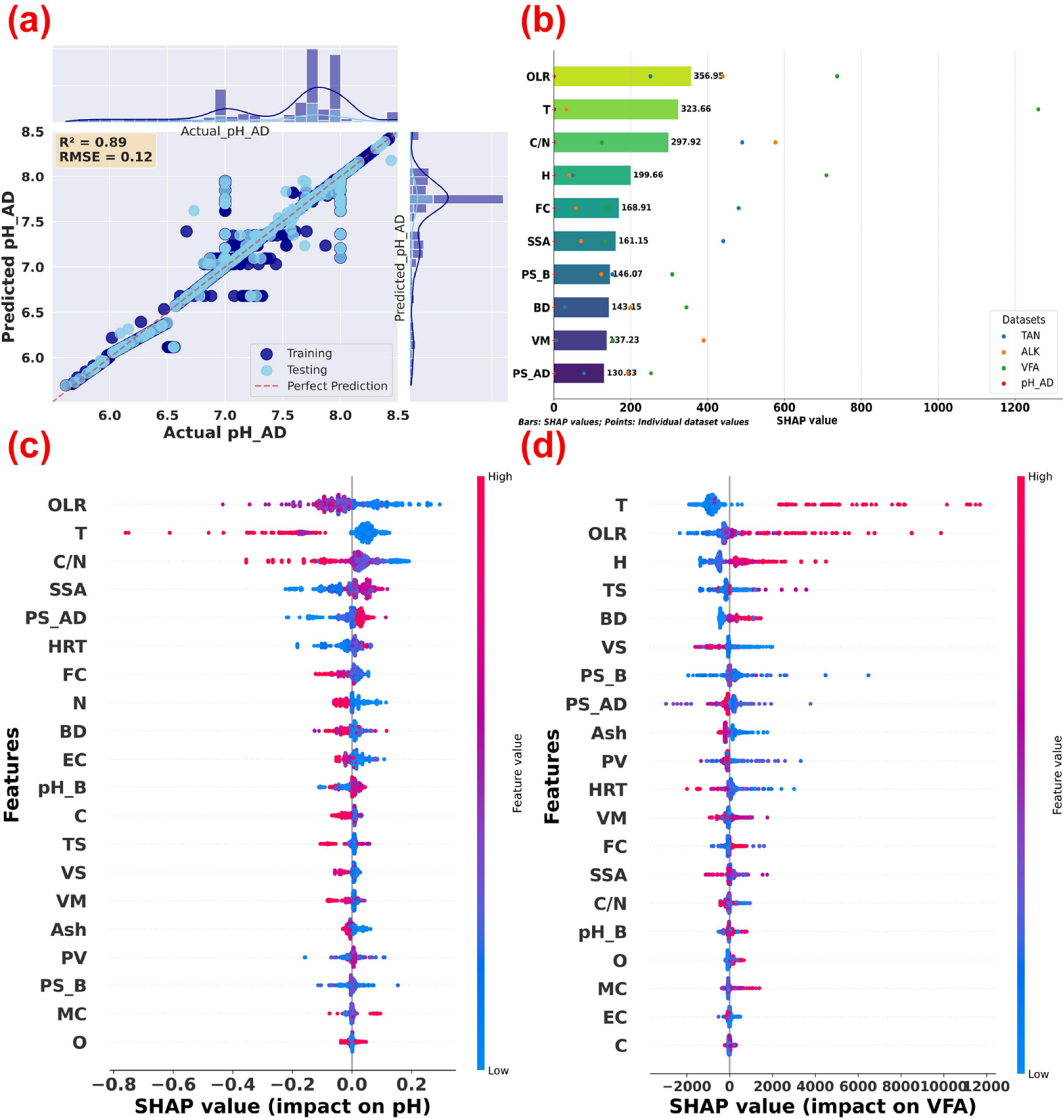
Interrelationships between anaerobic digestion (AD) system stability indicators and methane production. (a) Regression plot of digester pH. (b) Ranking of average SHAP importance values of top ten parameters. (c) SHAP summary plot of pH. (d) SHAP summary plot of volatile fatty acid (VFA). (AD: Anaerobic digestion; OLR: Organic loading rate; HRT: Hydraulic retention time; T: Temperature; C/N: Carbon/nitrogen ratio of AD feedstock; VS: Volatile solids content of AD feedstock; TS: Total solids content of AD feedstock; PS_AD: Particle size of AD feedstock; BD: Biochar dosage; SSA: Specific surface area of biochar; PV: Pore volume of biochar; pH_B: pH of biochar; PS_B: Particle size of biochar; VM: Volatile matter; FC: Fixed carbon; MC: Moisture content; C: Carbon content in biochar; H: Hydrogen; N: Nitrogen; O: Oxygen; pH_AD: Digester pH; VFA: Volatile fatty acid; ALK: Alkalinity; and TAN: Total ammonia nitrogen).

OLR emerged as a critical parameter influencing multiple process stability indicators, particularly digester pH (mean SHAP value: 356.95) and VFA concentrations. At high OLRs, the risk of VFA accumulation is also high, leading to unstable digester pH, which can inhibit methanogenic activity and reduce SMY.^61^ Additionally, high OLRs can result in increased TAN levels, which further destabilize the AD process, causing a significant decline in methane production. However, biochar’s buffering and adsorption capacities can mitigate these negative effects by stabilizing pH and adsorbing excess VFAs and TAN, ultimately enhancing process stability and SMY.^62^

Digester temperature emerged as a critical factor in maintaining digester stability, significantly impacting three important stability parameters, e.g., digester pH, VFA concentration, and ALK. This observation aligns with the previous studies highlighting the influence of digester temperature in regulating microbial community structure, VFAs and TAN dynamics, and overall methane yield.^63,64^ Thus, the effect of biochar augmentation on AD system performance also depends on system operating temperature. Thermophilic conditions (50–65 °C) accelerate biochemical reactions and microbial activity compared to mesophilic conditions (30–45 °C), leading to faster VFAs production and consumption. However, thermophilic AD systems are also more prone to instability due to lower microbial diversity and higher sensitivity to perturbations such as organic overloading, VFA accumulation, and ammonia toxicity.^65^ In contrast, mesophilic AD systems maintain a more stable and diverse microbial community, thereby reducing the risks of system acidification and process failure. Consequently, in general, biochar augmentation has shown more effectiveness in ensuring digester stability and improving methane production at thermophilic conditions than mesophilic conditions.^66–68^ AD process enhancement is attributed to biochar’s role in buffering the system, facilitating microbial immobilization, enabling DIET, and adsorbing excess TAN and VFAs. However, the performance of biochar in thermophilic AD system is not universally consistent across all conditions. Shen et al. reported that while biochar improved methane yield in mesophilic digesters, the effect was less pronounced in thermophilic digesters, especially when biochar derived from pine and white oak was used.^65^ This suggests that the effectiveness of biochar also depends on feedstock type and pyrolysis conditions, which influence its surface chemistry, porosity, and electrochemical properties.^65^

The C/N ratio emerged as a critical parameter influencing all four stability indicators: digester pH, VFA concentrations, ALK, and TAN levels, with its impact on TAN levels being particularly noteworthy. A low C/N ratio can result in excessive ammonia generation, potentially inhibiting methanogenic activity, while a high C/N ratio may limit nitrogen availability and hinder microbial growth.^69,70^ The dataset encompassed diverse AD feedstocks, ranging from nitrogen-rich animal manures to carbon-rich crop residues, resulting in a complex, nonmonotonic relationship between the C/N ratio and stability indicators.

Thus, depending on biochar type, augmented biochar can serve as both a nitrogen source^52^ and an ammonia sink,^71^ addressing issues associated with feedstocks that have high or low C/N ratios. In case of feedstocks with low C/N ratio, biochar, particularly high-porosity biochar with oxygen-containing functional groups, can adsorb and buffer ammonia, reducing its toxicity and promoting a stable methanogenic community.^71,72^ Studies have demonstrated that biochar’s adsorptive and electrochemical properties also support the development of ammonia-tolerant microbial communities, thereby improving methane production.^45,62,71^ For feedstocks with a high C/N ratio, nitrogen-rich biochar serves as a slow-release nitrogen source, supporting microbial growth and enhancing methane production.^10,52^ Thus, the effectiveness of biochar in AD systems depends on both feedstock characteristics and biochar properties, highlighting the importance of tailored biochar selection depending on the intended goal of biochar amendment.

Biochar characteristics, particularly hydrogen content, fixed carbon content, and specific surface area, demonstrated significant impacts on AD system stability indicators. Biochar with high specific surface area and high sorption capacity promotes microbial colonization and aids in alleviating toxicity caused by elevated VFAs and TAN levels.^73,74^ However, excessive biochar addition can negatively affect AD systems by potentially altering nitrogen availability and increasing pH level, which promotes the conversion of ammonium (NH_4_^+^) to its more toxic form, un-ionized ammonia (NH_3_). These effects are highly dependent on TAN concentration and biochar type, highlighting the need for careful selection of biochar type and its dosage to optimize methane production while preventing adverse effects.^71^

### 3.4 Model-Based Optimization for Guiding AD Experiments

The optimization framework developed in this study yielded key insights into the intricate interplay of parameters governing the performance of biochar-augmented AD systems. Based on the ten best optimization runs, optimal ranges for critical operational parameters were identified using a comprehensive optimization approach (Figure 5a). The results identified the optimal operational conditions as follows: OLR spanning from 2.5 to 4.8 g VS/L·d, HRT ranging from 19 to 46 days, the C/N ratio within 19.5–35.4, and temperature between 35.5 and 54.2 °C. These findings validated the results from interpretability analysis, which identified OLR as a key factor influencing SMY. Furthermore, the optimization process underscored the significance of biochar properties, particularly electrical conductivity and pore volume, in promoting SMY. The observed optimal ranges aligned closely with the interrelationships between AD system stability indicators and methane production (discussed in Section 4). By integrating stability considerations into the optimization framework, the proposed conditions ensure not only maximum methane yield but also enhanced AD system stability.

**Figure 5:**
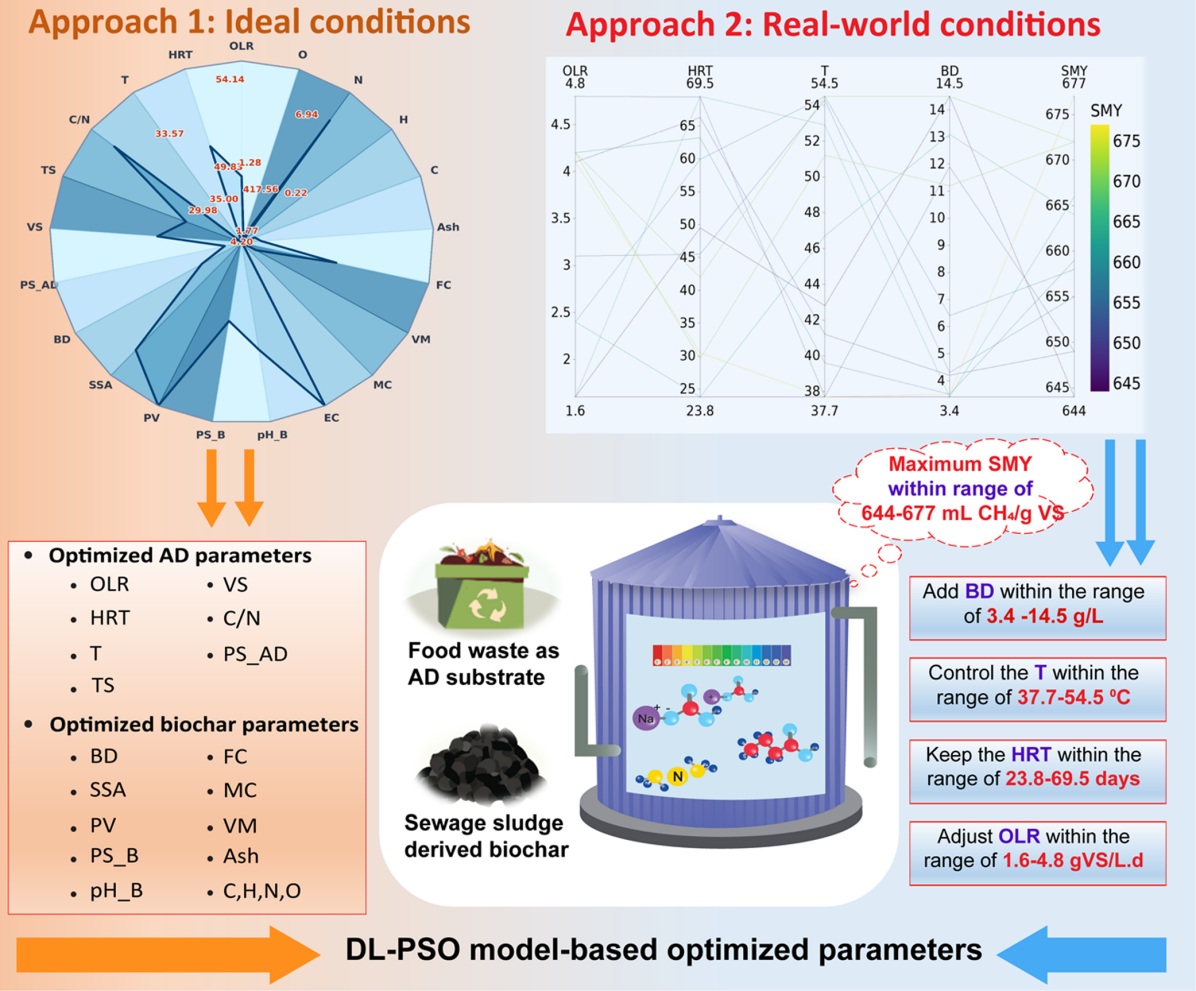
Model-based optimization results. (AD: Anaerobic digestion; OLR: Organic loading rate; HRT: Hydraulic retention time; T: Temperature; C/N: Carbon/nitrogen ratio of AD feedstock; VS: Volatile solids content of AD feedstock; TS: Total solids content of AD feedstock; PS_AD: Particle size of AD feedstock; BD: Biochar dosage; SSA: Specific surface area of biochar; PV: Pore volume of biochar; pH_B: pH of biochar; PS_B: Particle size of biochar; VM: Volatile matter; FC: Fixed carbon; MC: Moisture content; C: Carbon content in biochar; H: Hydrogen; N: Nitrogen; O: Oxygen; pH_AD: Digester pH; VFA: Volatile fatty acid; ALK: Alkalinity; and TAN: Total ammonia nitrogen).

To bridge the gap between theoretical optimization and real-world application, a second optimization approach was developed, focusing on controllable operational parameters. For this method, food waste as the AD feedstock and sewage sludge-derived biochar as an additive were selected, with fixed properties representing typical compositions encountered in practical AD system applications. The food waste characteristics include TS = 27.6%, VS = 95.0% (of TS), C/N ratio = 16.8, and particle size = ≤4.0 mm. The sewage sludge-derived biochar was characterized by specific surface area = 93 m^2^/g, pore volume = 0.2 cm^3^/g, particle size = 1.8 mm, pH = 8.9, electrical conductivity = 190 mS/cm, moisture content = 4.0%, volatile matter = 15.1%, fixed carbon = 12.5%, ash content = 84%, carbon content = 25.5%, hydrogen content = 0.1%, nitrogen content = 4.2%, and oxygen content = 0.6%. This real-world scenario allowed the optimization framework to focus on practical operational parameters. The ten best optimization runs identified optimal ranges of OLR (1.4–4.8 g VS/L·d), HRT (23.8–69.5 days), digester pH (7.9–8.0), temperature (37.7–54.5 °C), and biochar dosage (3.4–14.5 g/L). These results demonstrated the potential for achieving high SMY (644–677 mL CH_4_/g VS) even with fixed feedstock properties, providing a practical roadmap for optimizing biochar-augmented AD systems in diverse operational settings (Figure 5b). By using feedstock and biochar characteristics that are representative of real-world situations, this approach bridges the gap between theoretical findings and practical applications. The results offer valuable insights for researchers and practitioners aiming to optimize AD systems for process stability with enhanced methane yield from similar organic waste streams.

## 4 Limitations

Despite the strong predictive performance of the developed stacking ensemble DL model, several limitations should be acknowledged. The dataset primarily represents laboratory-scale biochar-augmented AD systems, which, while ensuring internal consistency, limit direct extrapolation to pilot- or industrial-scale operations. Factors such as mixing heterogeneities, mass transfer limitations, reactor hydrodynamics, and long-term biochar stability, which are more pronounced at large scales, were not captured by the current model.

Additionally, although random splits, five-fold cross-validation, and external dataset validation were implemented to enhance robustness, the possibility of overlapping operating conditions (e.g., temperature, HRT, biochar dosage) between training and testing subsets may have contributed to optimistic performance estimates. While consistent results across validation scenarios (e.g., low variation in *R* ^2^) suggest model stability, future work should apply study-wise cross-validation to minimize overlap and better assess generalizability to untested datasets. The model was developed based on experimental conditions within specific parameter ranges, and its predictions outside these ranges remain untested. Validation on additional datasets incorporating different feedstocks, biochar types, and operational conditions will be essential to confirm model reliability for broader applications.

A further limitation of this study is the absence of direct microbial community data, reflecting the broader scarcity of standardized microbial datasets in biochar-augmented AD studies. A review of the literature indicates that only a limited number of studies report quantitative microbial data alongside operational and physicochemical parameters. Where such data are available, methodological heterogeneity—including differences in 16S rRNA sequencing, qPCR, metagenomics, taxonomic resolution, and reporting formats—poses challenges for standardization and integration into ML frameworks.^75–77^ While recent studies have shown that incorporating microbial community data can enhance the predictive accuracy of AD models,^27,75,78^ future efforts in biochar-augmented AD system modeling would benefit from the inclusion of standardized microbial metrics. Achieving this will require consistent protocols for microbial data collection, analysis, and reporting to support the development of more sophisticated AI/ML models capable of capturing the complex biochar–microbe interactions that drive system performance. Finally, the optimal operational parameter ranges identified in this study are based on model-driven global optimization results and have not yet been validated through controlled experiments. Targeted validation experiments at representative points within the identified optimal ranges are recommended to empirically verify model predictions and refine operational guidance for real-world implementation. Addressing these limitations—through expanded datasets, integration of pilot- and full-scale data, inclusion of microbial metrics, empirical validation of optimized conditions, and simplification of model architecture—will help bridge the gap between laboratory-scale insights and practical industrial implementation.

## 5 Implications and Perspectives

Our interpretable stacking ensemble DL model advances biochar-augmented AD optimization by integrating comprehensive datasets that capture complex interactions between feedstock characteristics, operational parameters, biochar properties, and stability indicators. The model achieved exceptional predictive accuracy (*R* ^2^ of 0.94, RMSE of 61 mL CH_4_/g VS), demonstrating how hybrid DL architectures can effectively model complex biological processes where traditional mechanistic models fall short. To further assess the generalizability of our model beyond the original training and internal validation datasets, an external validation set consisting of ten independent lab-scale samples drawn from previously published AD studies (details provided in Tables S1 and S4) was compiled. These samples represented diverse substrates, biochar types, and operational conditions that were not present in the model development data, offering a rigorous test of model's generalizability. Importantly, the external samples were entirely distinct from those used during model training and internal validation. When applied to this external dataset, the developed stacking ensemble DL model achieved a mean *R* ^2^ of 0.68 (Figure S17), demonstrating its ability to predict methane yield under unseen lab-scale conditions and supporting its broader applicability to biochar-augmented AD system optimization.

The model-based global optimization framework simultaneously optimized operational conditions and biochar characteristics, delivering 15–20% higher methane production compared to nonoptimized systems, significantly enhancing energy recovery and economic viability. A user-friendly GUI makes this technology accessible to researchers working on biochar-augmented AD systems, with validation confirming prediction errors consistently below 10% (Figure S13 in the Supporting Information). From an environmental sustainability perspective, optimized biochar-augmented AD systems offer benefits beyond enhanced methane production. The ability to operate efficiently at high OLRs enables more compact waste treatment with a small footprint, reducing the capital cost of AD facilities.^9^ Additionally, the integration of biochar represents a carbon sequestration strategy, as the recalcitrant carbon remains stable after AD, potentially creating a carbon-negative waste management pathway.^79,80^

This study opens several promising avenues for future research. Expanding the dataset to include pilot- and industrial-scale systems, diverse feedstocks, and biochar types will enhance model generalizability and practical relevance. Integrating standardized microbial community data could provide deeper mechanistic insight into biochar–microbe interactions and support targeted bioaugmentation strategies. The incorporation of real-time monitoring data would enable dynamic optimization under fluctuating conditions, improving resilience in full-scale applications.^81^ Future efforts should also focus on simplifying the model to reduce computational demands and facilitate faster deployment. Additionally, this methodological framework could be extended to other waste-to-energy technologies and environmental applications of biochar.

## Supplementary Material



## Data Availability

Codes related to this paper can be found on the Open Science Framework (https://osf.io/rmeqx/?view_only=9d2ebc54d3e2470e81c21b8740a92e69, see Files tab).

## Notes

Detailed raw data analysis, data sources, comprehensive model development, evaluation, and interpretability, an in-depth description of global optimization, additional hyperparameter optimization, and the GUI interface (PDF)
