# Supplementary material for: Biochar-Augmented Anaerobic Digestion System: Insights from an Interpretable Stacking Ensemble Deep Learning
Source: Environ Sci Technol. 2025 Jul 18;59(29):15236–50. doi: 10.1021/acs.est.5c05051 (PMC12312089; doi:10.1021/acs.est.5c05051)
Supplement: Supplementary file 1 [file es5c05051_si_001.pdf]

Biochar-Augmented Anaerobic Digestion System: Insights from an Interpretable Stacking Ensemble Deep Learning

Muzammil Khan[a,b,c 1], K. C. Surendra[a 1], Sachita Baniya[a,b], Jay Rhymer[d], Samir Kumar Khanal[a,b,e,f *]

[a]Department of Molecular Biosciences and Bioengineering (MBBE), University of Hawaiʻi at Mānoa, 1955 East-West Road, Honolulu, HI 96822, USA.

[b]Department of Civil, Environmental and Construction Engineering (CECE), University of Hawaiʻi at Mānoa, 2540 Dole Street, Honolulu, HI 96822, USA.

[c]School of Engineering and Energy, College of Science, Technology, Engineering & Mathematics, Murdoch University, 90 South Street, Murdoch, WA 6150, Australia

[d]Punahou School, 1601 Punahou St, Honolulu, HI 96822, USA

[e]Department of Civil and Environmental Engineering, The Hong Kong University of Science and Technology, Clear Water Bay, Kowloon, Hong Kong, China

[f]Affiliate faculty, Department of Environmental Engineering, Korea University Sejong Campus, Sejong-ro 2511 Sejong 2511, Korea

*Corresponding author: cekhanal@ust.hk; khanal@hawaii.edu (S. K Khanal)

[1]The authors contributed equally to this work.

This file includes 43 pages (S1–S45), 8 supplementary texts (Text S1–S8), 19 supplementary figures (Figure S1–S19), and 5 supplementary tables (Table S1–S5)

Contents:

Text S1:	Dataset Detailed Description	S3–S4
Text S2:	Correlation Analysis	S4–S5
Text S3:	Preliminary Analysis of Raw Dataset	S5–S7
Text S4:	Brief Overview of Deep Learning Models Development and Evaluation	S7–S8
Text S5:	Detailed Description of Hyperparameter Optimization	S9
Text S6:	Predictive Performance Evaluation of Deep Learning Models	S9–S10
Text S7:	Interpretability Analysis of Deep Learning Models	S10–S11
Text S8:	Model-Based Optimization Framework – PSO-Based Memetic Algorithm	S11–S12
Figure S1:	Violin and correlation plots for preliminary data analysis	S13
Figure S2:	Training and validation loss comparison of all deep learning models	S14
Figure S3:	CNN hyperparameters scatterplot matrix	S15
Figure S4:	LSTM hyperparameters scatterplot matrix	S16
Figure S5:	CNN-LSTM series hyperparameters scatterplot matrix	S17
Figure S6:	LSTM-CNN series hyperparameters scatterplot matrix	S18
Figure S7:	CNN-LSTM parallel hyperparameters scatterplot matrix	S19
Figure S8:	Extra Tree Regressor hyperparameters scatterplot matrix	S20
Figure S9:	Optimized hyperparameters of various models	S21
Figure S10:	Prediction performance of 38 different meta-learner machine-learning models	S22
Figure S11:	Residual plots comparison of all deep learning models	S23
Figure S12:	Regression plots showing actual vs predicted specific methane yield (SMY) for base and stacking ensemble deep learning (DL) models	S24
Figure S13:	Stacking ensemble deep learning (DL) model deployment and validation	S25
Figure S14:	Predictive performance of various deep learning models using repeated 5-fold cross-validation (5 repeats).	S26
Figure S15:	Training and validation loss comparison for all deep learning models across each of the 5 folds in cross-validation.	S27
Figure S16:	Predictive performance of various deep learning models using repeated 5-fold cross-validation (5 repeats).	S28
Figure S17:	External validation of the stacking ensemble model on 10 biochar-augmented anaerobic digestion (AD) samples	S29
Figure S18:	Frequency distribution of anaerobic digestion substrates used across studies.	S30
Figure S19:	Variations of specific methane yield (SMY) predicted by stacking ensemble DL model over 100 experimental data points	S31
Table S1:	Sources of biochar-augmented anaerobic digestion dataset	S32–S34
Table S2:	Raw dataset ranges collected from experimental studies	S35
Table S3:	Optimized hyperparameters	S36
Table S4	Summary of external validation dataset	S37
Table S5	Summary of machine learning-based studies on biochar-augmented anaerobic digestion processes	S37

Text S1: Dataset Detailed Description.

An extensive literature search was conducted to collect experimental data on biochar-augmented semi-continuous anaerobic digestion (AD) systems. The search was performed using Google Scholar and Web of Science, employing a broad set of keywords such as "biochar-added," "biochar-enhanced," "biochar-augmented," "semi-continuous AD," "biogas production," "methane production," and "VFA production." The selection focused specifically on studies using continuous stirred-tank reactors (CSTRs) in semi-continuous mode due to their widespread application in commercial AD processes. To ensure the reliability and relevance of the dataset, a rigorous screening process was implemented. This involved eliminating duplicate entries, review articles, and book chapters while retaining only original research articles. Following this process, 26 peer-reviewed studies published between 2018 and 2024 were selected. These studies examined a diverse range of 15 different organic waste types, including food waste, sewage sludge, and agricultural residues, as well as 10 different biochar types derived from various sources such as wood waste and herbaceous materials.

Given the heterogeneous nature of the collected data, preprocessing steps were undertaken to ensure data consistency and reliability. To address this issue, different approaches were employed depending on the dataset characteristics. For the SMY-Dataset, where missing values were minimal, the MissForest imputation method was used to fill in gaps while preserving the integrity of variable relationships. [1,2] MissForest is a non-parametric, iterative imputation method based on Random Forests. [3] It does not assume a specific distribution for the data, making it highly effective in handling both numerical and categorical variables. The method operates by first initializing missing values using the mean (for continuous variables) or mode (for categorical variables). Then, a Random Forest model is trained iteratively to predict missing

values based on observed data. This process continues until convergence is achieved, meaning that the imputed values remain stable across iterations. The advantage of MissForest is that it captures complex relationships between variables, ensuring a more accurate and unbiased imputation process. For the Stability Indicator-Dataset, where missing values were more substantial, data points with excessive missing values were removed to maintain model reliability. Following imputation and filtering, all data were standardized to ensure consistency across different studies.

Text S2: Correlation Analysis

Pearson's correlation coefficient (PCC): PCC is a statistical measure that quantifies the strength and direction of a linear relationship between two continuous variables. [4] It is calculated using the formula:

$$r = \frac{\sum(x_i - \bar{x})(y_i - \bar{y})}{\sqrt{\sum(x_i - \bar{x})^2 \sum(y_i - \bar{y})^2}}$$

r	=	Pearson correlation coefficient
x_i	=	values of the x-variable in a sample
$\bar{x}$	=	mean of the values of the x-variable
y_i	=	values of the y-variable in a sample
$\bar{y}$	=	mean of the values of the y-variable

PCC ranges from -1 to +1, where +1 indicates a perfect positive correlation, -1 signifies a perfect negative correlation, and 0 denotes no correlation.

Spearman's rank correlation coefficient (SRC): SRC is a non-parametric measure that assesses the strength and direction of a monotonic relationship between two variables, meaning it detects both linear and non-linear trends. [4] It is calculated using the formula:

$$\rho = 1 - \frac{6\sum d_i^2}{n(n^2 - 1)}$$

ρ	=	Spearman's rank correlation coefficient
d_i	=	difference between the two ranks of each observation
n	=	number of observations

SRC ranges from -1 to +1, similar to Pearson's, but is particularly useful when the relationship between variables is not strictly linear.

Text S3: Preliminary Analysis of Raw Dataset

Figure S1a illustrates the distribution of key variables in biochar-augmented AD systems. Biochar properties exhibited wider distributions compared to AD parameters, reflecting diverse biochar feedstocks and production conditions. Biochar's specific surface area ranged from nearly 2.6 to over 500 m^2/g (median ~200 m^2/g), which indicates substantial differences in microbial colonization potential. [5,6] Carbon content varied considerably between 5.6% and 82.1%, with high-carbon biochar typically derived from woody biomass and low-carbon biochar associated with nutrient-rich feedstocks such as animal manure and sewage sludge. [7,8] Biochar tends to exhibit alkaline pH values (7–11), suggesting a potential buffering effect in AD systems, [9] while electrical conductivity varied widely from 0.01 to 280 mS/cm, potentially influencing nutrient availability and microbial activity. [9] In contrast, AD parameters showed narrower distributions, indicating tighter process control. Digester temperature exhibited a bimodal distribution, clearly delineating mesophilic and thermophilic conditions. HRT ranged from 2 to 80 days, while OLR

varied between 0.6 and 20.0 g VS/L.d, reflecting diverse experimental setups. The C/N ratio ranged widely from 6.5 and 35.4 (mean 17.7), encompassing both nitrogen-limited and ammonia toxicity conditions. SMY showed a broad range of 20–1375 mL CH_4/g VS, with notable upper outliers (≥500 mL CH_4/g VS), underscoring the complex interplay between biochar properties and AD system parameters.

Both Pearson (r) and Spearman rank (ρ) correlation coefficient analysis revealed the strongest correlation between VS and TS (r = 0.6416, ρ = 0.7982) (Figure S1b). This relationship suggested that TS could serve as a reliable predictor of VS, crucial for estimating biodegradable content. [10] Biochar's carbon and ash contents exhibited a strong negative correlation (r = -0.7396, ρ = -0.5650), indicating an inverse relationship critical for understanding biochar's composition and its potential impact on SMY. Notably, ash content showed consistently strong negative correlations with oxygen content (r = -0.7272, ρ = -0.7395) and volatile matter content (r = -0.7010, ρ = -0.6796) across both methods. Ash content showed strong negative correlations with oxygen content (r = -0.7272, ρ = -0.7395) and volatile matter content (r = -0.7010, ρ = -0.6796). This suggests that the non-ash components of biochar (volatile matter and carbon structures containing oxygen functional groups) play an important role in biogas production, though the optimal ash content likely depends on specific feedstock characteristics and operating conditions. [11,12] Biochar's hydrogen content demonstrated consistent positive correlations with oxygen content (r = 0.6288, ρ = 0.5990) and volatile matter (r = 0.6268, ρ = 0.5889). Interestingly, the relationships between biochar moisture and nitrogen content (ρ = -0.5706) and between substrate particle size and biochar specific surface area (ρ = 0.5459) were prominent in the spearman analysis but less so in the PCC analysis. Such discrepancy highlighted the presence of monotonic but not necessarily linear relationships in the biochar-augmented AD process,

emphasizing the importance of considering non-linear interactions when optimizing AD process for SMY. The dendrogram analysis highlighted key variable clusters, such as the strong interconnections among biochar's total carbon, fixed carbon, and volatile matter contents, as well as the grouping of digester temperature, HRT, and OLR, indicating their collective influence on AD performance. These insights can inform process optimization strategies, guide feedstock selection, and contribute to developing more accurate models for predicting and optimizing AD system performance.

Text S4: Brief Overview of Deep Learning Models Development and Evaluation

To explore the full potential of advanced deep learning (DL) models, our study employs a variety of CNN and LSTM architectures, [13,14] both standalone and hybrid, to model the complex dynamics of biochar-enhanced AD process.

Standalone models: We first developed a standalone CNN model to focus on extracting spatial features from the input data. [13,14] This model leverages convolutional layers to identify patterns and relationships within the biochar and AD operational parameters. Next, we developed a standalone LSTM model designed to capture temporal dependencies. [13,14] This model utilizes recurrent layers to process sequences of data points, excelling in scenarios where understanding the temporal evolution of the AD process, such as changes in methane production over time, is critical.

Sequential architectures: We then developed a sequential architecture, CNN-LSTM in series, where the feature extraction capabilities of CNNs are leveraged first, followed by LSTMs to model the temporal dependencies of these features. Another sequential architecture we developed is LSTM-CNN in series, which begins with an LSTM to capture temporal patterns

and sequences initially. Subsequently, a CNN processes these temporal features to extract feature relationships. This approach is suited for data where time-dependent patterns, such as the progression of AD stages, influence spatial feature representations.

Parallel architectures: We also developed a parallel architecture where both CNN and LSTM networks process the input data concurrently. Their outputs are then merged, typically through concatenation, to combine spatial and temporal insights effectively. This architecture is ideal for applications requiring rapid processing and integration of complex, multi-dimensional data, such as simultaneous analysis of biochar properties and AD operational conditions.

Stacking ensemble architecture: To further improve performance, we employed stacking ensemble architectures which combines multiple models to improve overall performance by leveraging the strengths of each previously developed individual model (CNN, LSTM, CNN-LSTM in series, LSTM-CNN in series, and parallel CNN-LSTM). The stacking ensemble integrates the outputs of several diverse models using a meta-model to learn the final prediction. This ensemble method effectively reduces the variance and bias of individual models, leading to more reliable predictions. The ensemble approach is particularly beneficial in scenarios where combining multiple perspectives can lead to a more comprehensive understanding and improved predictive performance.

Text S5: Detailed Description of Hyperparameter Optimization

To ensure that each DL model operates at its optimal capacity, we employed hyperparameter optimization using the Optuna framework. [13,14] Traditional trial-and-error methods for tuning hyperparameters are not only inefficient but also often fail to identify the best

configurations due to their subjective nature and limited exploration of the hyperparameter space. In contrast, Optuna provides a robust and efficient approach by dynamically constructing the search space based on ongoing trials, a technique known as the define-by-run approach. [13,14] This allows for a more informed and adaptive optimization process, enabling systematic and efficient hyperparameter tuning. Optuna supports both single-objective and multi-objective optimization, which is particularly beneficial for complex deep learning tasks where multiple performance metrics need to be optimized simultaneously. This ensures that our models achieve not only high accuracy but also a well-balanced trade-off between various performance criteria. Another key advantage of Optuna is its ability to handle conditional hyperparameters, meaning it can optimize parameters that depend on the values of other hyperparameters. This feature significantly enhances the flexibility of the optimization process, allowing for more precise tuning of DL architectures. A major strength of Optuna is its smooth integration with major DL frameworks, including TensorFlow, PyTorch, and scikit-learn. This compatibility facilitates easy incorporation into our existing workflows, enabling systematic exploration of the hyperparameter space.

Text S6: Predictive Performance Evaluation of Deep Learning Models

To quantifiably evaluate the performance of these models, the root means square error (RMSE) and the coefficient of determination (R^2) were calculated and compared. [15] The equations for these metrics are as follows:

$$R^2 = 1 - \frac{\sum_{i=1}^{m}(y_i - \hat{y}_i)}{\sum_{i=1}^{m}(y_i - \overline{y})}$$

$$RMSE = \left(\frac{\sum_{i=1}^{m}(y_i - \hat{y}_i)^2}{m}\right)^{1/2}$$

R^2 = Coefficient of determination

M = number of data points for training or testing datasets

y_i = ith observation

$\hat{y}_i$ = corresponding predicted value

$\bar{y}$ = average value of I data points

Text S7: Interpretability analysis of deep learning models

Permutation importance (PI): PI is a straightforward yet powerful method for assessing feature importance in machine learning and deep learning models. [16,17] This technique involves randomly shuffling the values of a single feature while keeping the others unchanged, then measuring the resulting change in model accuracy. If the model's performance deteriorates significantly after shuffling a feature, it indicates that the feature is highly important. Conversely, if there is little to no change in accuracy, the feature has a minimal impact on predictions. This approach provides a global ranking of feature importance, offering a simple way to identify the most influential parameters in the model.

Shapley Additive Explanations (SHAP): SHAP is a game-theoretic approach to explain the output of machine learning models. [18,19] It assigns each feature a SHAP value, representing its contribution to the model's prediction for a specific data point. SHAP is derived from cooperative game theory, where the contribution of each feature is evaluated based on all possible feature subsets. Unlike PI, which only provides a global ranking, SHAP allows for both local (individual prediction-level) and global (overall feature importance) interpretability.

Partial dependence plots (PDPs): PDPs illustrate the marginal effect of one or more input features on the predicted outcome by averaging the model's predictions over a range of values for the selected feature(s). One-way PDPs examine the effect of a single feature on target (SMY) while keeping all other features constant. Two-way PDPs explore interactions between two features simultaneously, providing deeper insights into how combinations of inputs (biochar properties and AD conditions) influence target (SMY). These were particularly useful in identifying nonlinear dependencies and synergistic effects between parameters.

Text S8: Model-based optimization framework – PSO-based memetic algorithm

Particle swarm optimization (PSO): PSO is a population-based metaheuristic optimization algorithm inspired by the collective movement of biological swarms, such as flocks of birds or schools of fish. [16,20] The algorithm optimizes a given objective function by iteratively improving candidate solutions based on their positions and velocities in the search space. Each candidate solution, referred to as a particle, adjusts its movement according to its own best-known position and the global best-known position of the swarm. PSO is well-known for its fast convergence and ability to navigate complex solution spaces, making it a suitable method for optimizing AD process parameters and biochar properties. However, PSO often struggles with premature convergence, where particles get trapped in local optima rather than exploring the broader solution space effectively. To address this limitation, a memetic algorithm was incorporated into the optimization framework.

Memetic algorithm (MA) and hybridization with PSO: MA is an enhanced evolutionary algorithm that integrates global search techniques (such as PSO or genetic algorithms) with local search methods (such as gradient-based optimization). [20,21] This hybridization ensures that while the global search mechanism explores the entire parameter space, a local search technique refines

the best solutions found, improving overall accuracy and convergence speed. In our framework, the memetic algorithm enhances PSO by applying gradient descent as a local optimizer. Once PSO identifies promising candidate solutions, gradient descent is employed to fine-tune these solutions by iteratively minimizing the error function. By incorporating gradient descent, the memetic algorithm corrects small deviations and ensures fine-tuned parameter selection, overcoming the tendency of PSO to converge prematurely. This hybrid approach balances exploration and exploitation, making it particularly effective for optimizing the multi-dimensional parameter space of biochar-augmented AD systems.

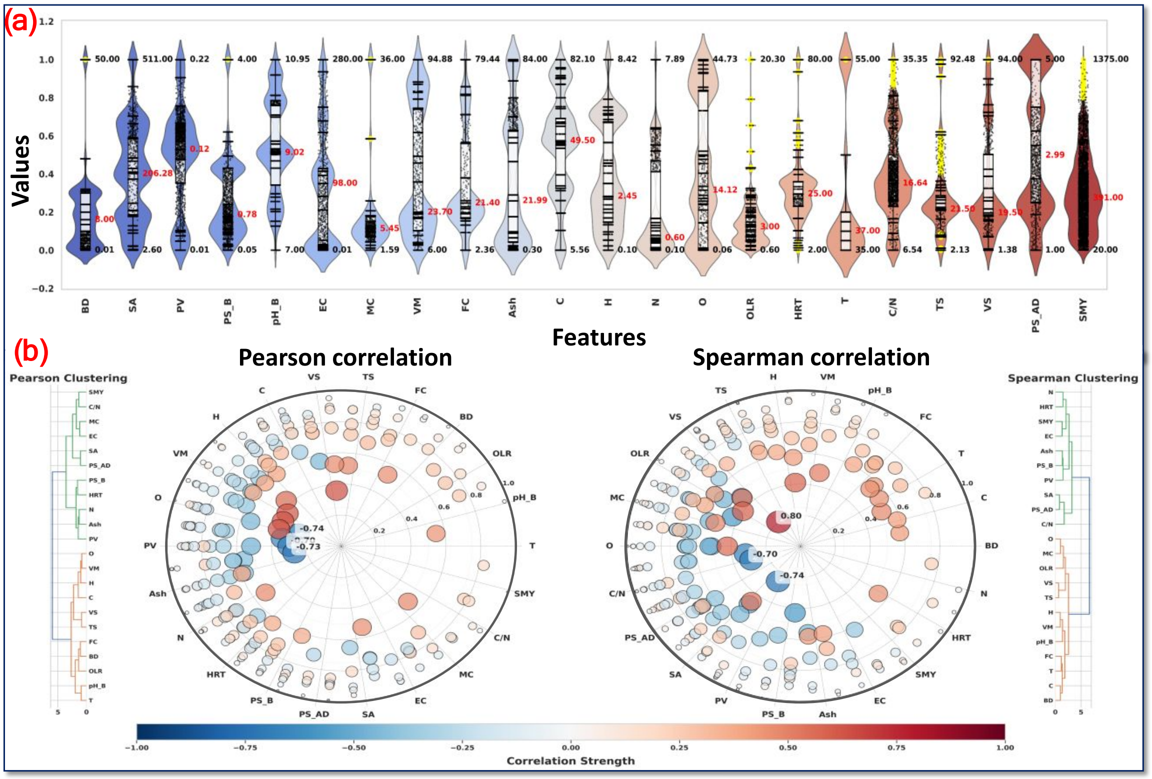

Figure S1: Violin and correlation plots for preliminary data analysis. (a) The outer shape of the violin represents the probability density of the data at different values, with wider sections indicating a higher probability of observations at that value and narrower regions representing lower probabilities. Overlaid on each violin is a box plot, where the white box represents the interquartile range (IQR), with the horizontal line inside the box indicating the median. The whiskers extension shows the rest of the distribution, excluding outliers. Individual data points are represented by small black dots, providing a view of the raw data distribution. Additionally, yellow dots represent outliers, and the actual minimum, maximum, and median values for each parameter are displayed alongside each violin. (b) The circular plots visually represent the strength and direction of correlations, with colors ranging from deep red (strong positive, +1) through white (no correlation, 0) to deep blue (strong negative, -1), and bubble size indicating correlation strength. Accompanying dendrograms illustrate the hierarchical clustering of variables based on their correlation patterns. (AD: Anaerobic digestion; OLR: Organic loading rate; HRT: Hydraulic retention time; T: Temperature; C/N: Carbon/nitrogen ratio of AD feedstock; VS: Volatile solid content of AD feedstock; TS: Total solid content of AD feedstock; PS_AD: Particle size of AD feedstock; BD: Biochar dosage; SSA: Specific surface area of biochar; PV: Pore volume of biochar; pH_B: pH of biochar; PS_B: Particle size of biochar; VM: Volatile matter; FC: Fixed carbon; MC: Moisture content; C: Carbon content ofin biochar; H: Hydrogen; N: Nitrogen; O: Oxygen; pH_AD: Digester pH ; VFA: Volatile fatty acids; ALK: Alkalinity; and TAN: Total ammonia nitrogen)

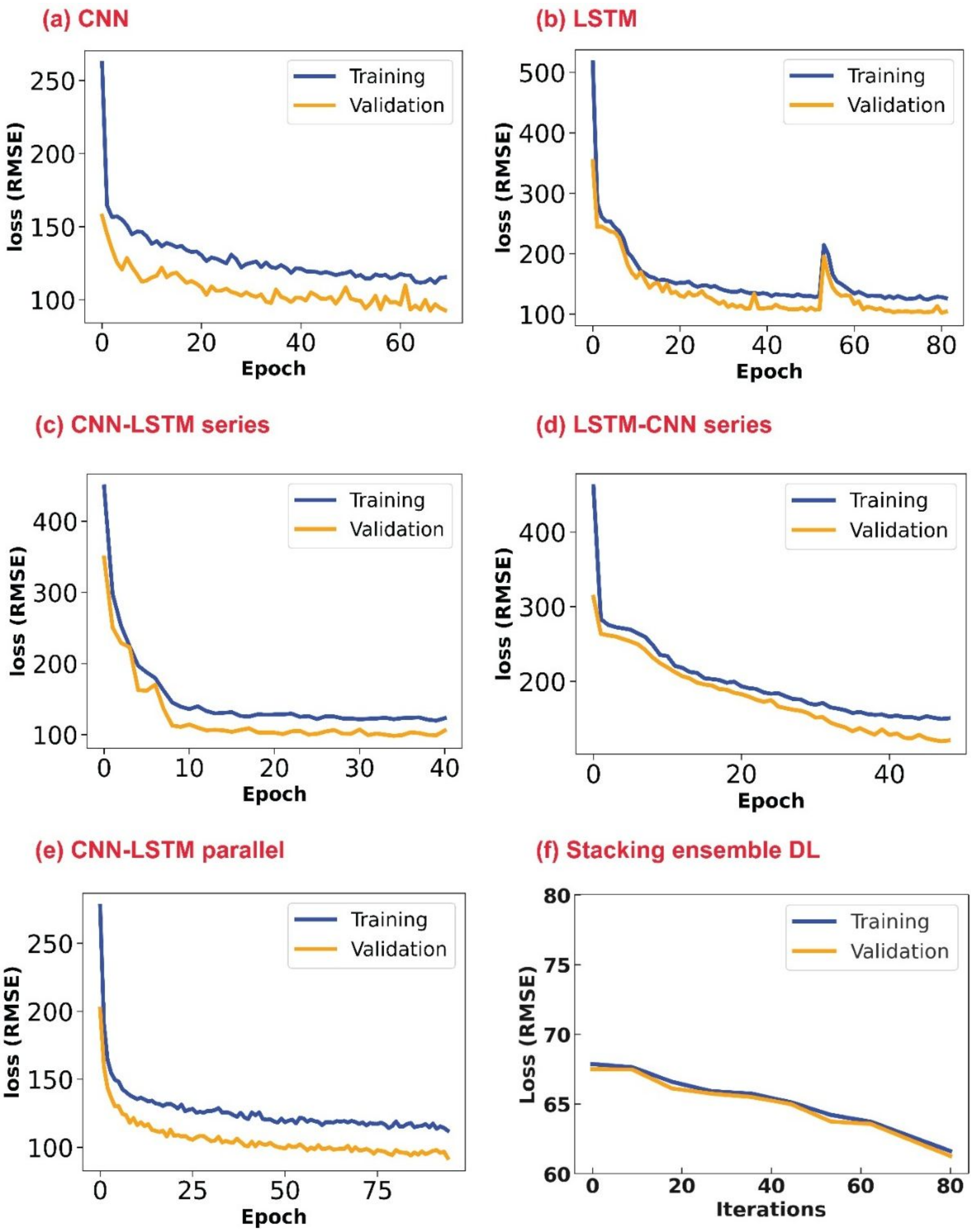

Figure S2: Training and validation loss comparison of all deep learning models

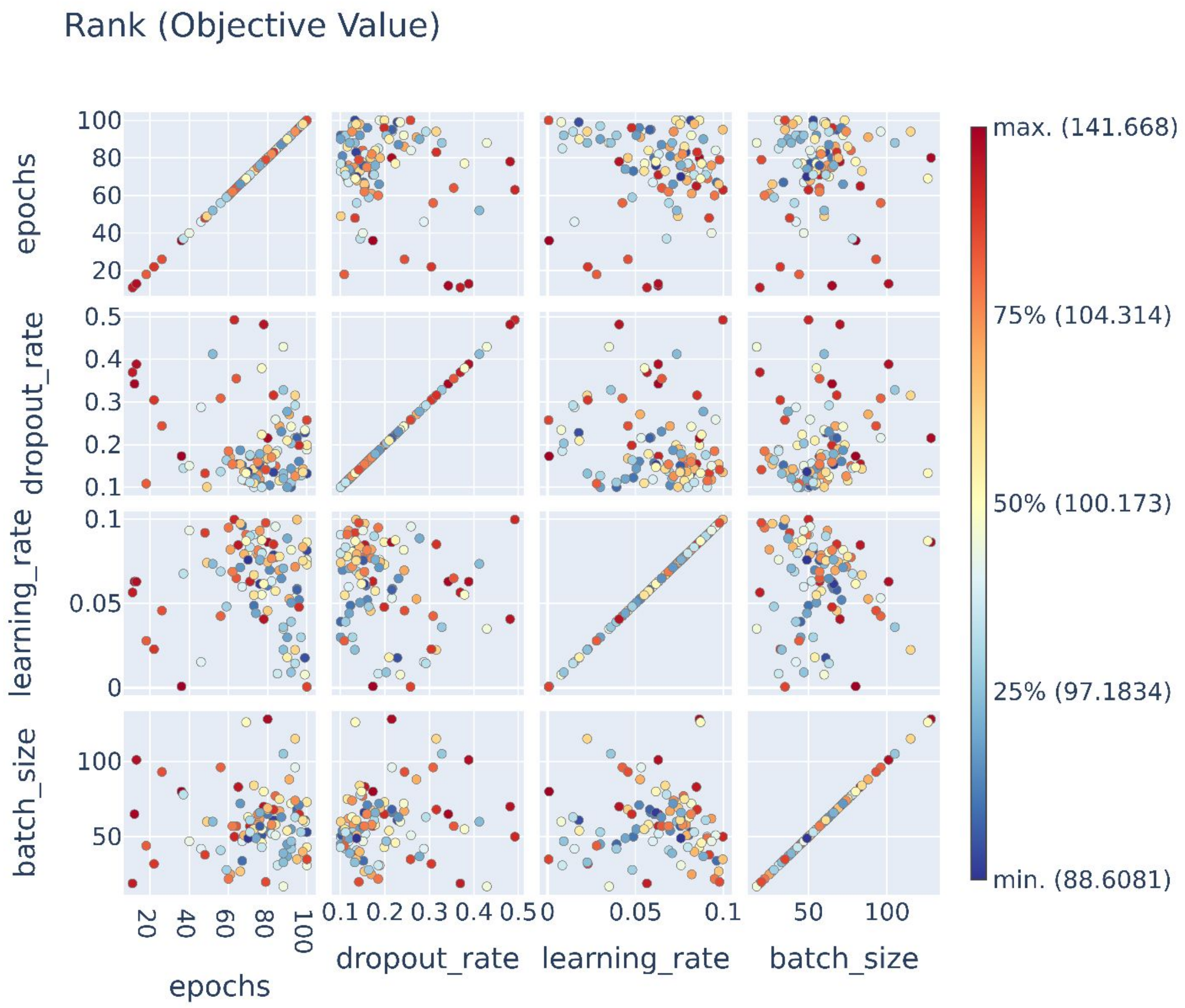

Figure S3: Scatterplot matrix visualizing the relationships between different hyperparameters of a convolutional neural network (CNN) and their objective value ranks (performance metric)

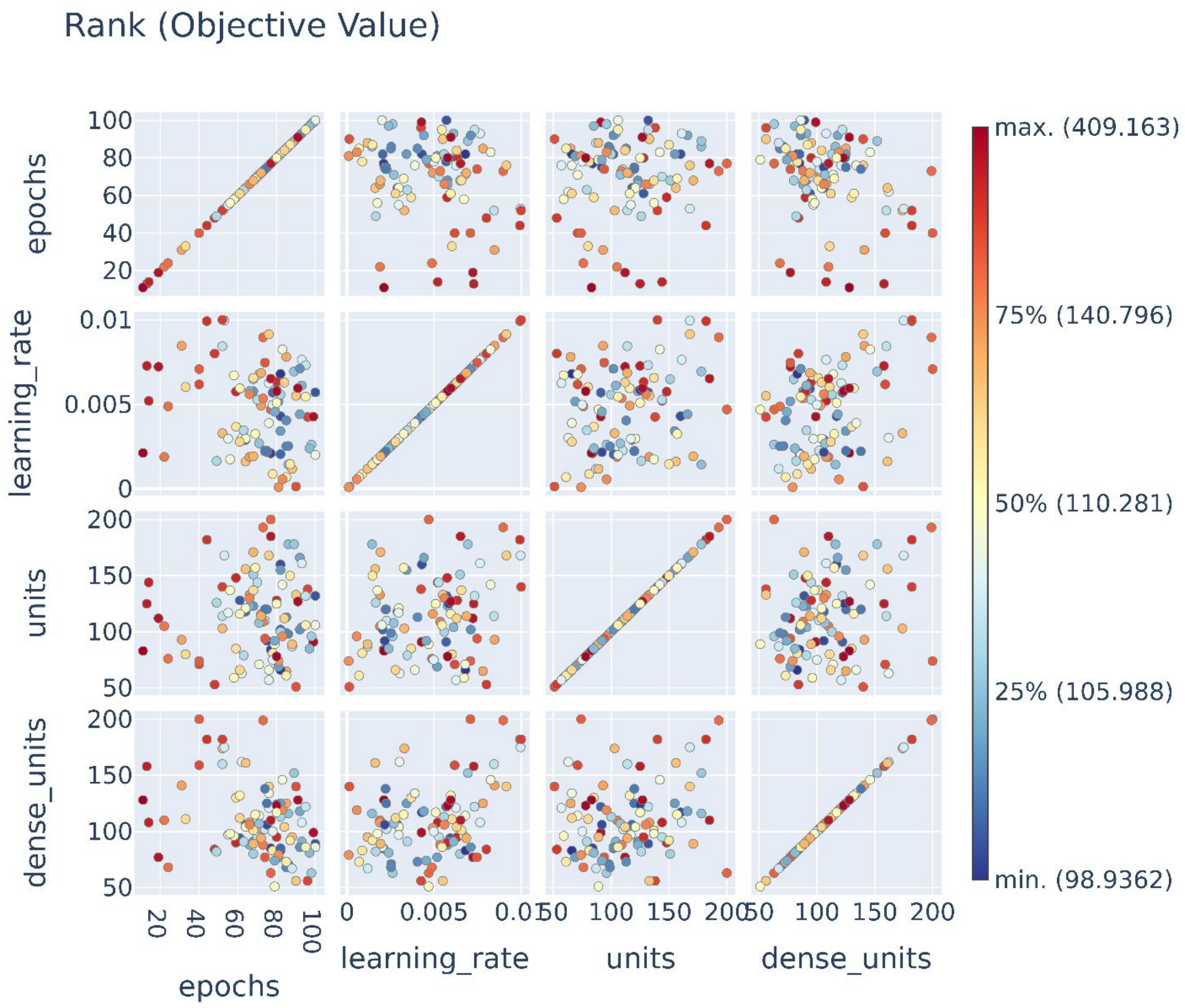

Figure S4: Scatterplot matrix visualizing the relationships between different hyperparameters of a Long short-term memory (LSTM) and their objective value ranks (performance metric).

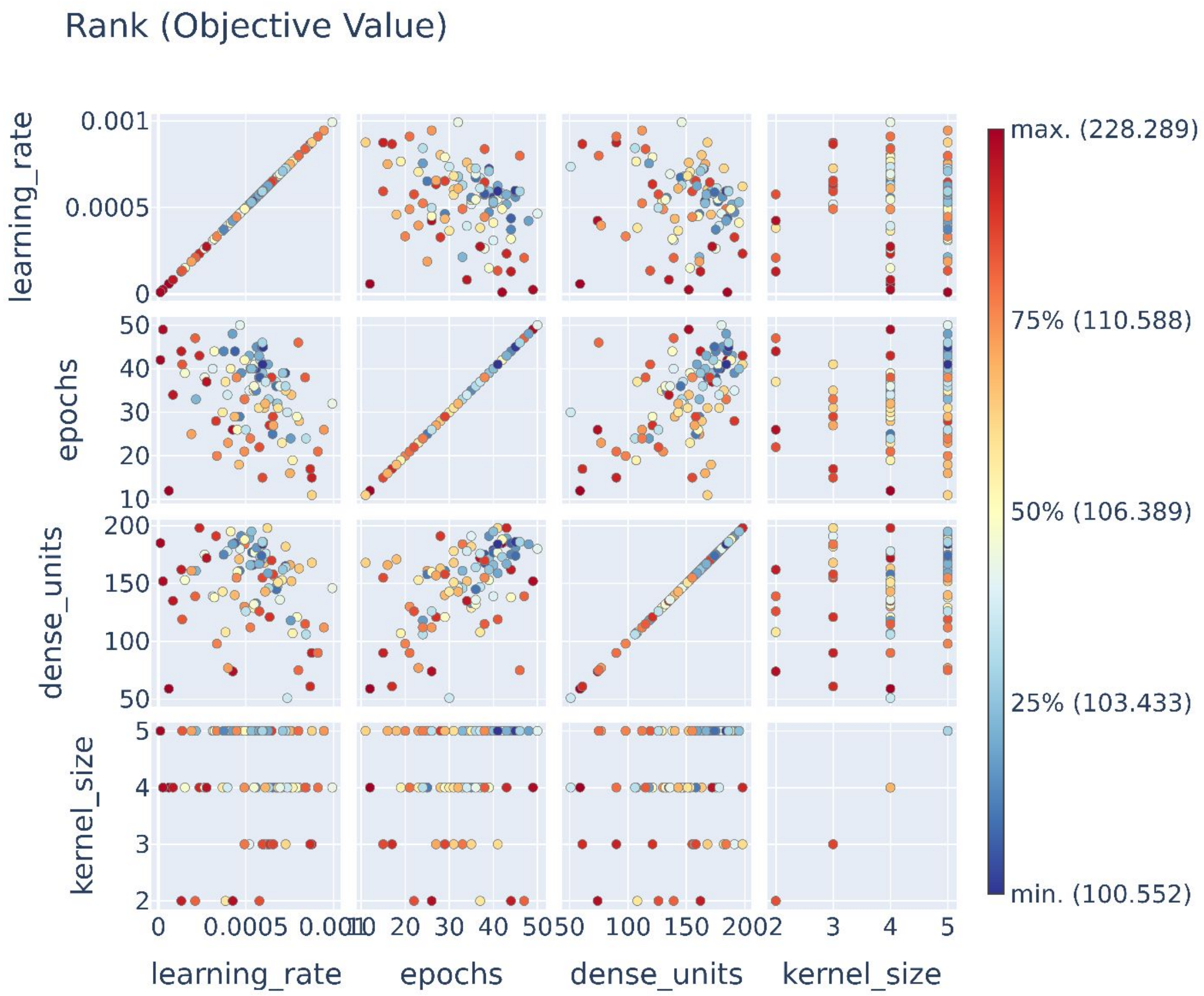

Figure S5: Scatterplot matrix visualizing the relationships between different hyperparameters of a convolutional neural network (CNN)-Long short-term memory (LSTM) series (CNN-LSTM series) and their objective value ranks (performance metric).

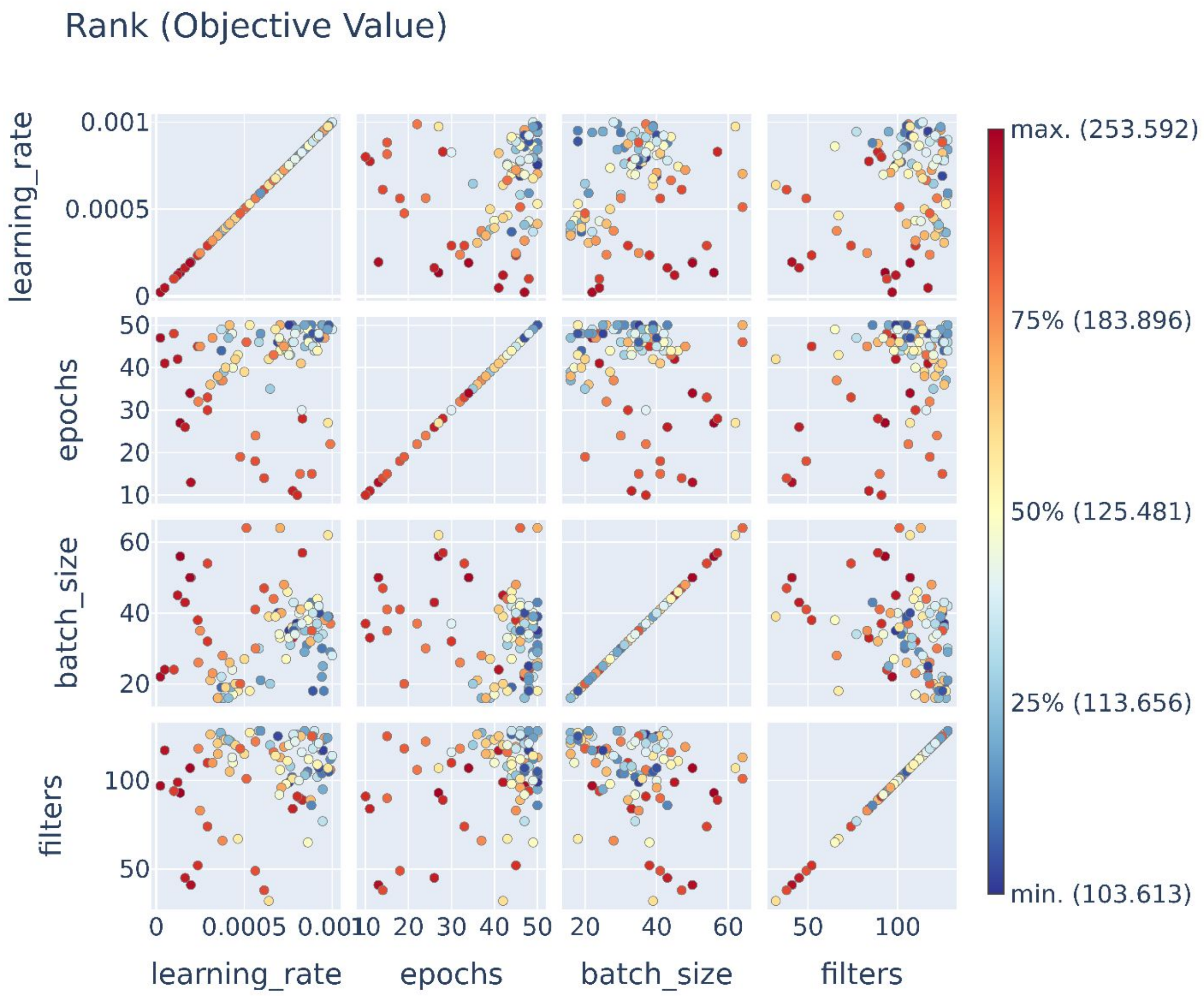

Figure S6: Scatterplot matrix visualizing the relationships between different hyperparameters of a Long short-term memory (LSTM)-convolutional neural network (CNN) series (LSTM-CNN series) and their objective value ranks (performance metric).

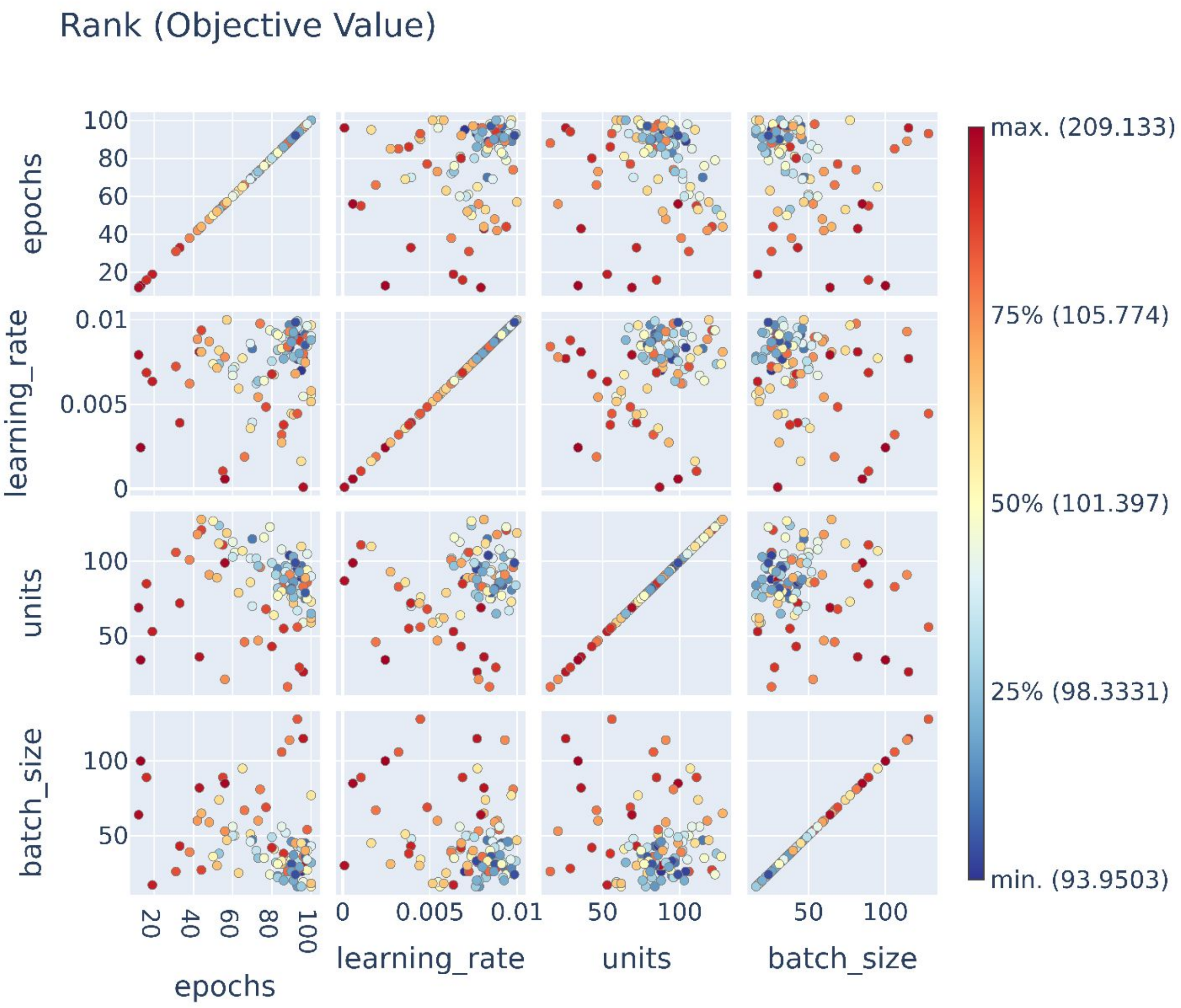

Figure S7*:* Scatterplot matrix visualizing the relationships between different hyperparameters of a convolutional neural network (CNN)-Long short-term memory (LSTM) parallel (CNN-LSTM parallel) and their objective value ranks (performance metric).

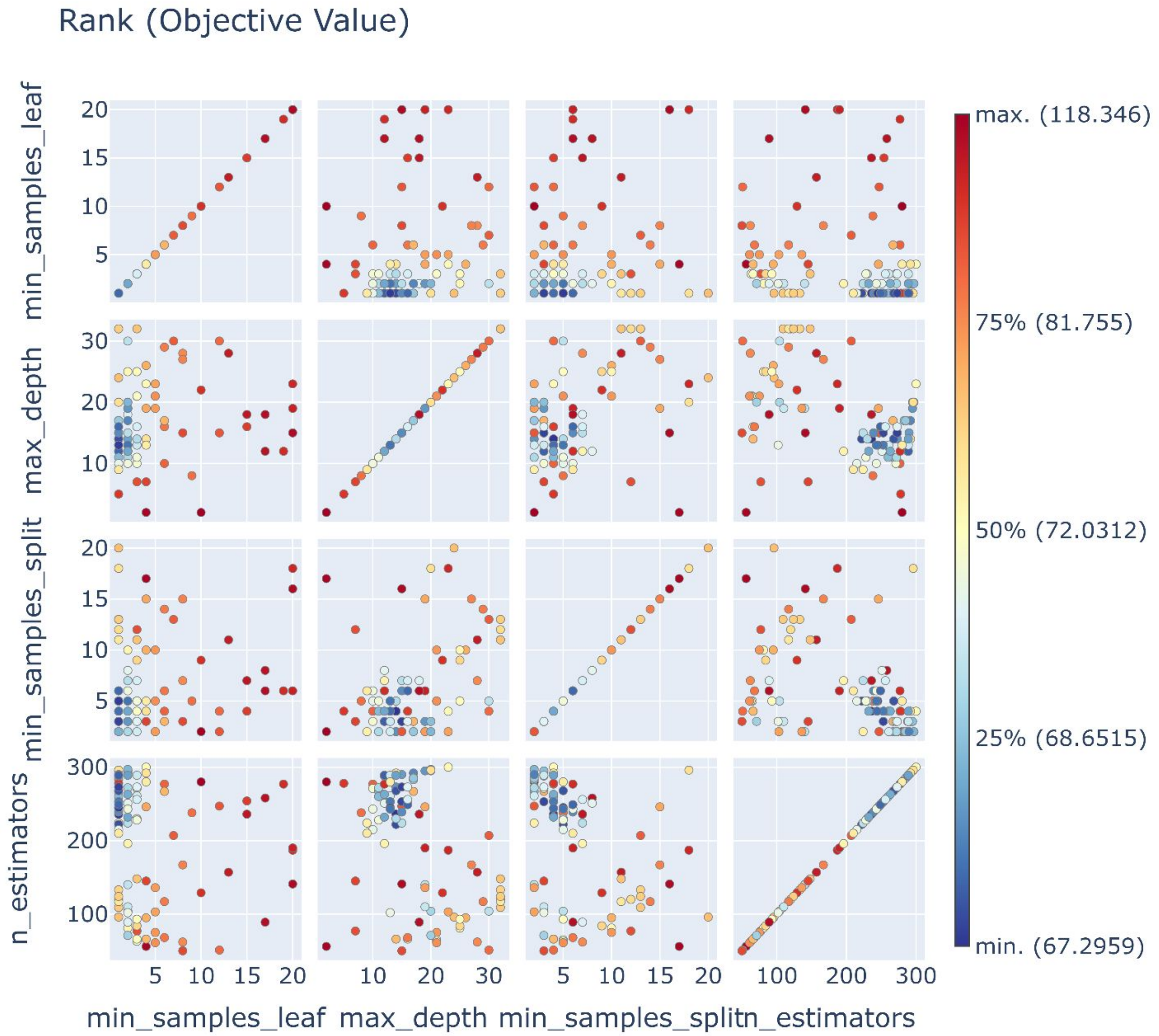

Figure S8: Scatterplot matrix visualizing the relationships between different hyperparameters of an Extra Tree Regressor model used in stacking ensemble deep learning model and their objective value ranks (performance metric).

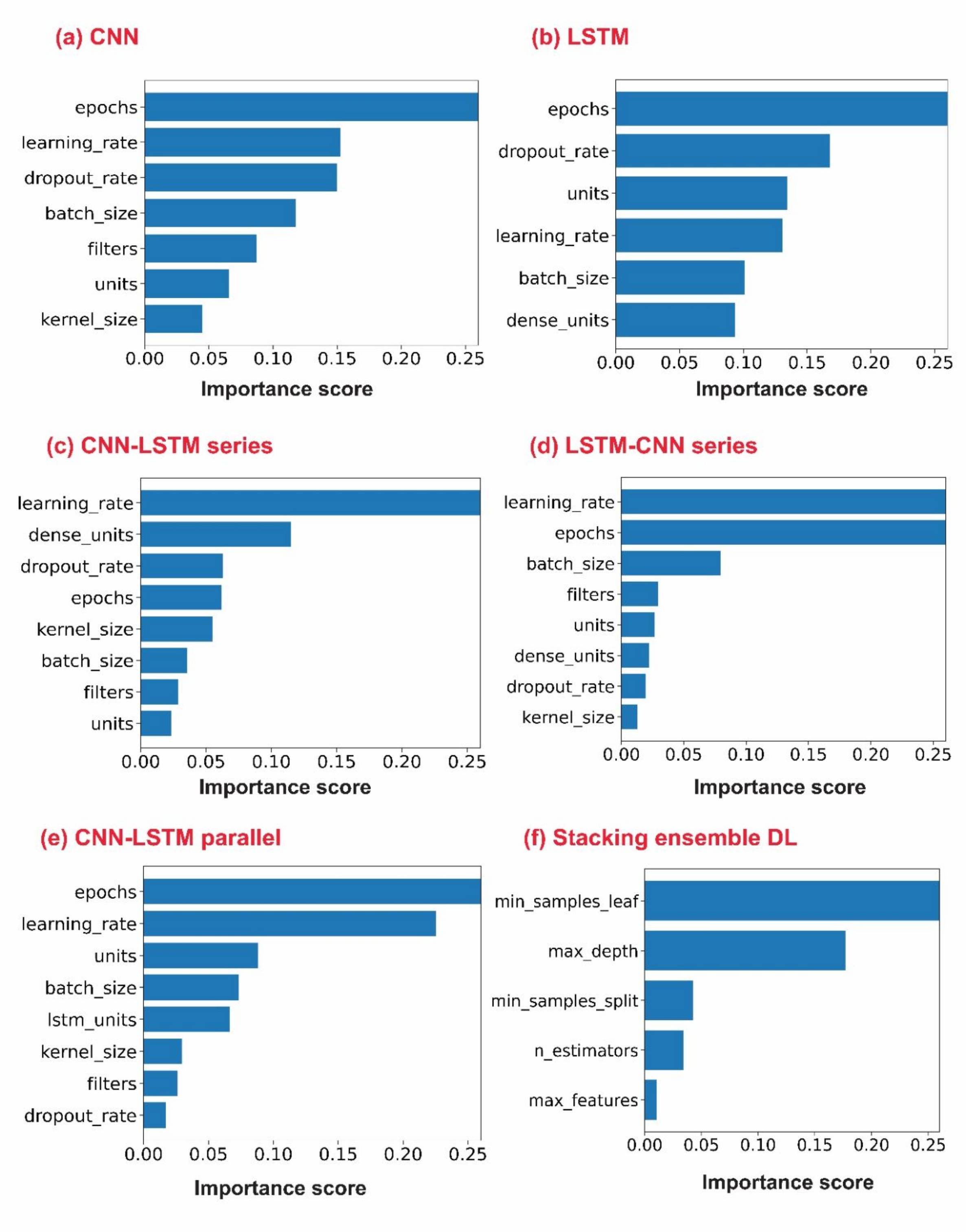

Figure S9: Optimized hyperparameters of various models including (a) convolutional neural network (CNN), (b) long short-term memory (LSTM), (c) CNN followed by LSTM in series (CNN-LSTM series), (d) LSTM followed by CNN in series (LSTM-CNN series), (e) CNN followed by LSTM in parallel (CNN-LSTM parallel), and (f) Extra tree regressor as meta learner in stacking ensemble deep learning (DL) model

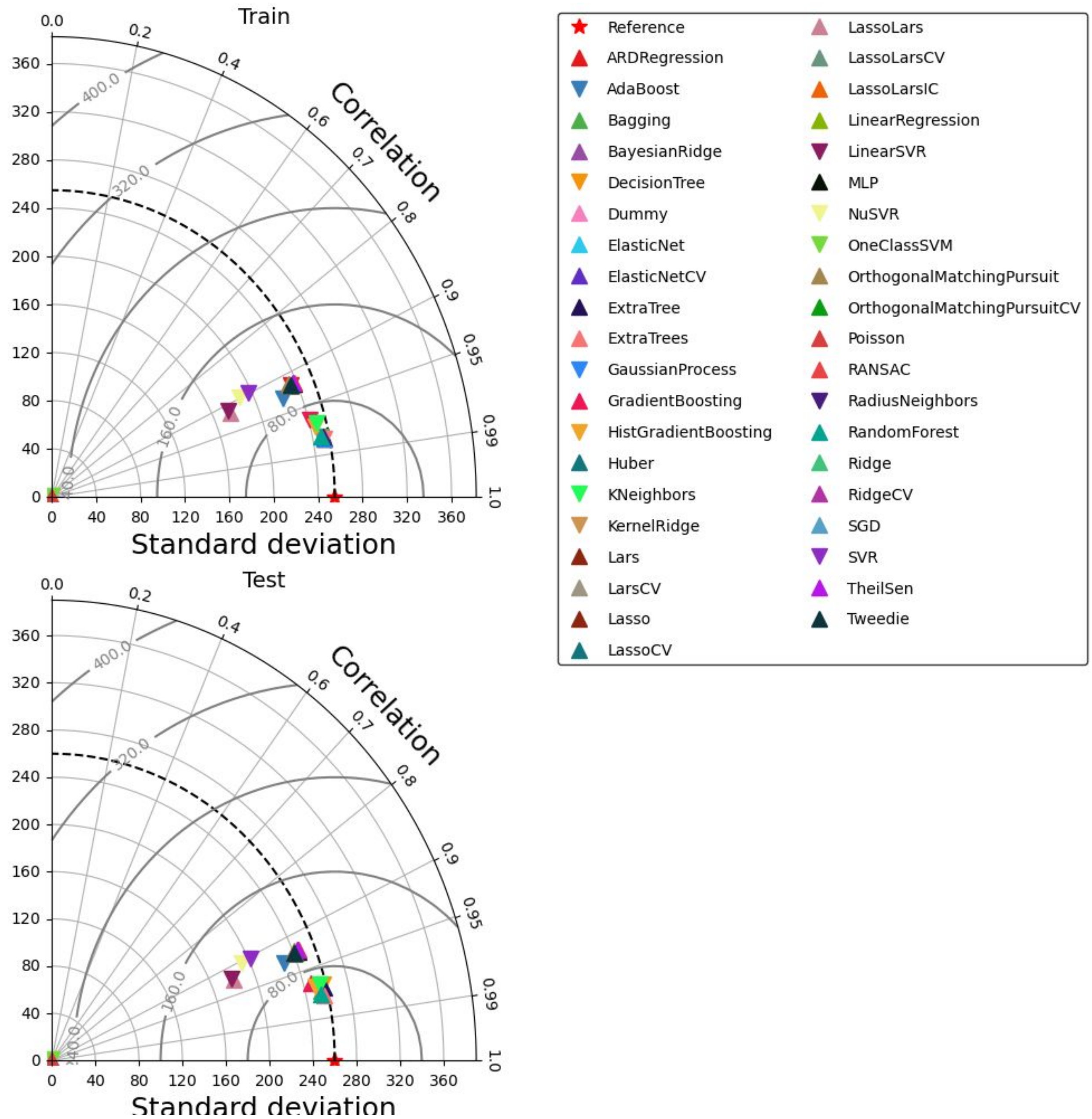

Figure S10: Prediction performance of 38 different machine-learning models used as meta-learners for stacking ensemble deep learning models

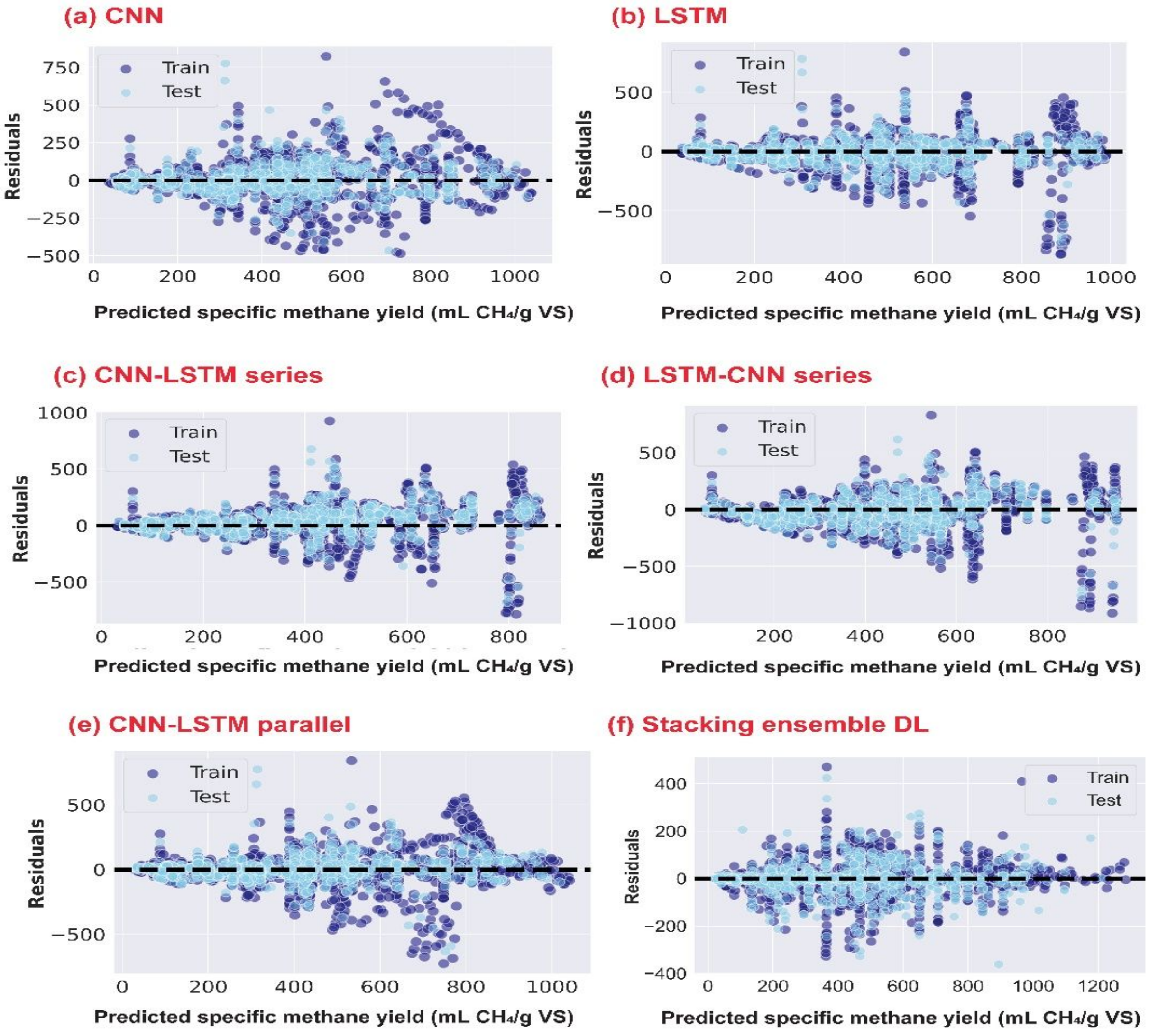

Figure S11: Residual plots comparison of all deep learning models. (a) convolutional neural network (CNN), (b) long short-term memory (LSTM), (c) CNN followed by LSTM in series (CNN-LSTM series), (d) LSTM followed by CNN in series (LSTM-CNN series), (e) CNN followed by LSTM in parallel (CNN-LSTM parallel), and (f) stacking ensemble deep learning (DL) model

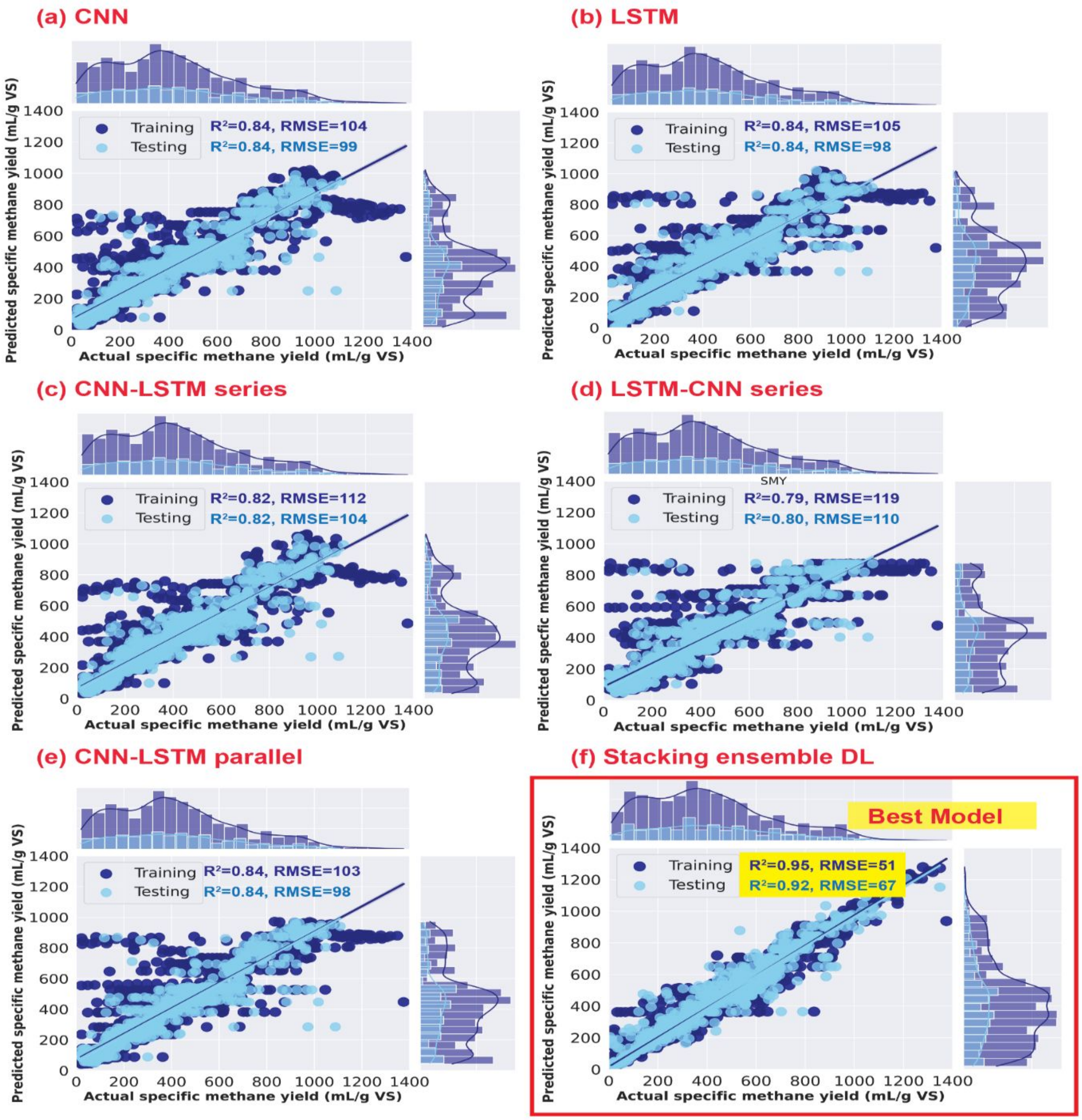

Figure S12: Regression plots showing actual vs predicted specific methane yield (SMY) for base and stacking ensemble deep learning (DL) models. (a) convolutional neural network (CNN), (b) long short-term memory (LSTM), (c) CNN followed by LSTM in series (CNN-LSTM series), (d) LSTM followed by CNN in series (LSTM-CNN series), (e) CNN followed by LSTM in parallel (CNN-LSTM parallel), and (f) stacking ensemble deep learning (DL) model

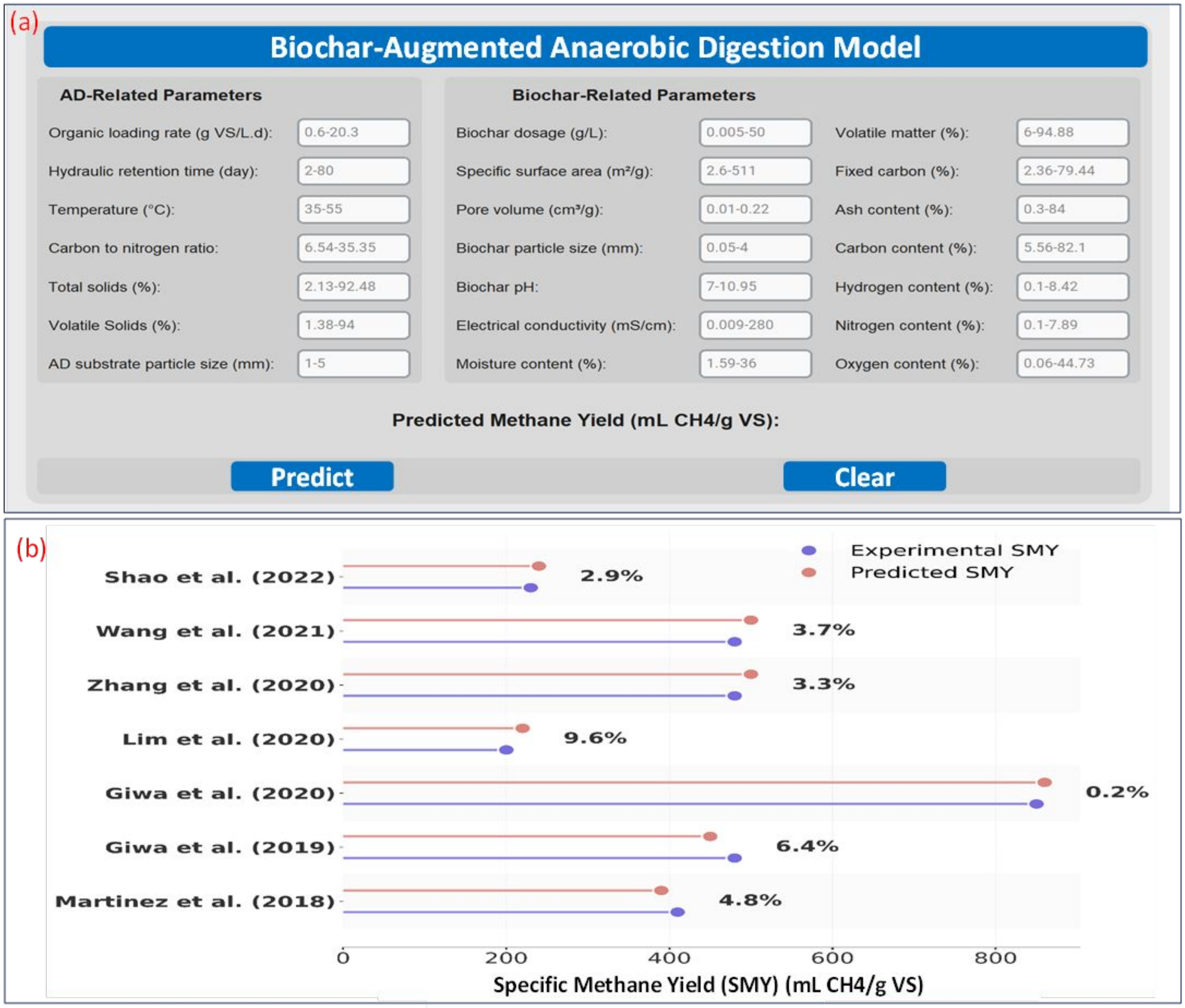

Figure S13: Stacking ensemble deep learning (DL) model deployment and validation. (a) Graphical user interface (GUI) for predicting specific methane yield (SMY) based on stacking ensemble DL model. (b) Validation of GUI using data from literature

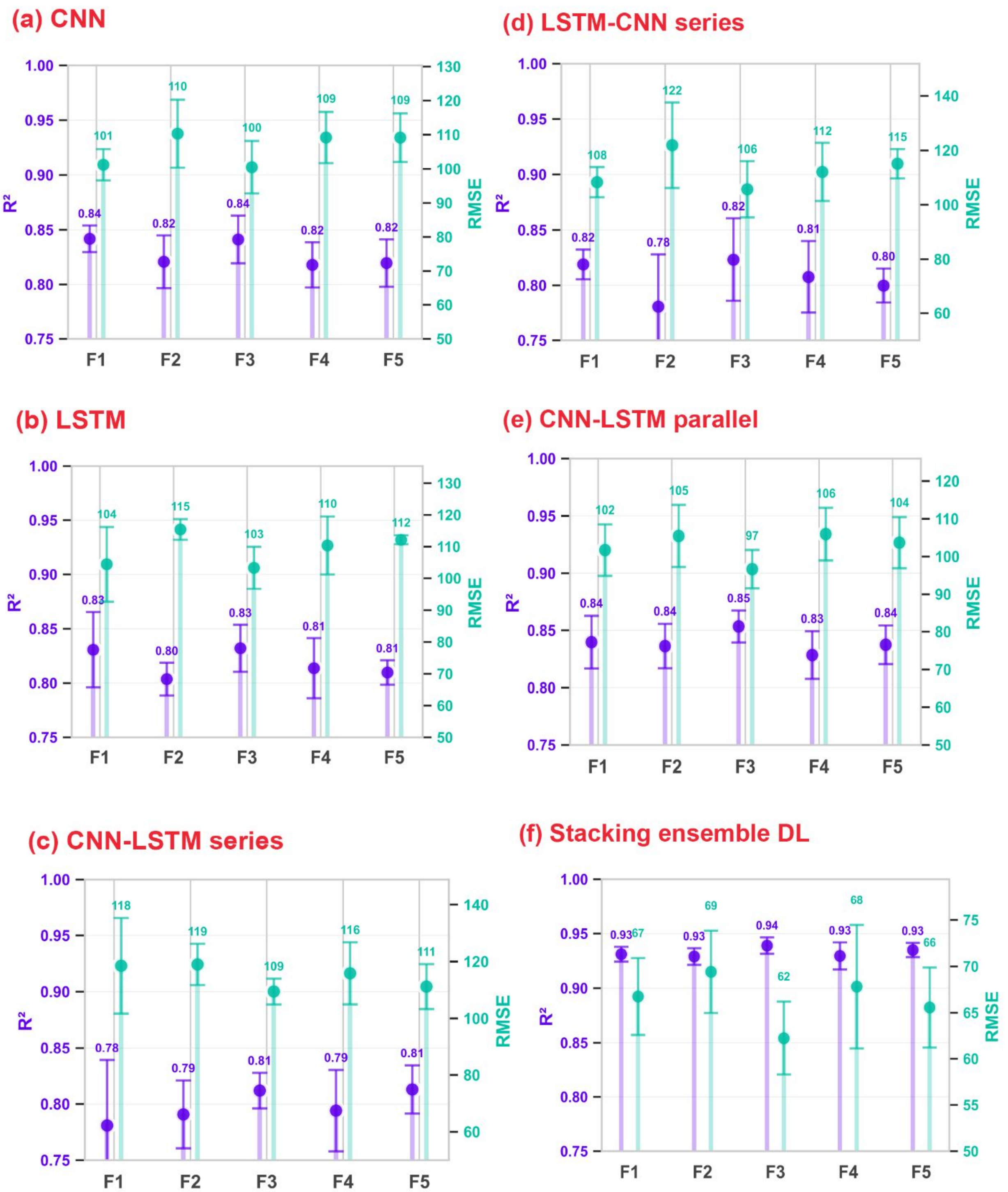

Figure S14: Predictive performance of various deep learning models using repeated 5-fold cross-validation (5 repeats). F1–F5 indicate the average performance for each fold across the 5 repeats.

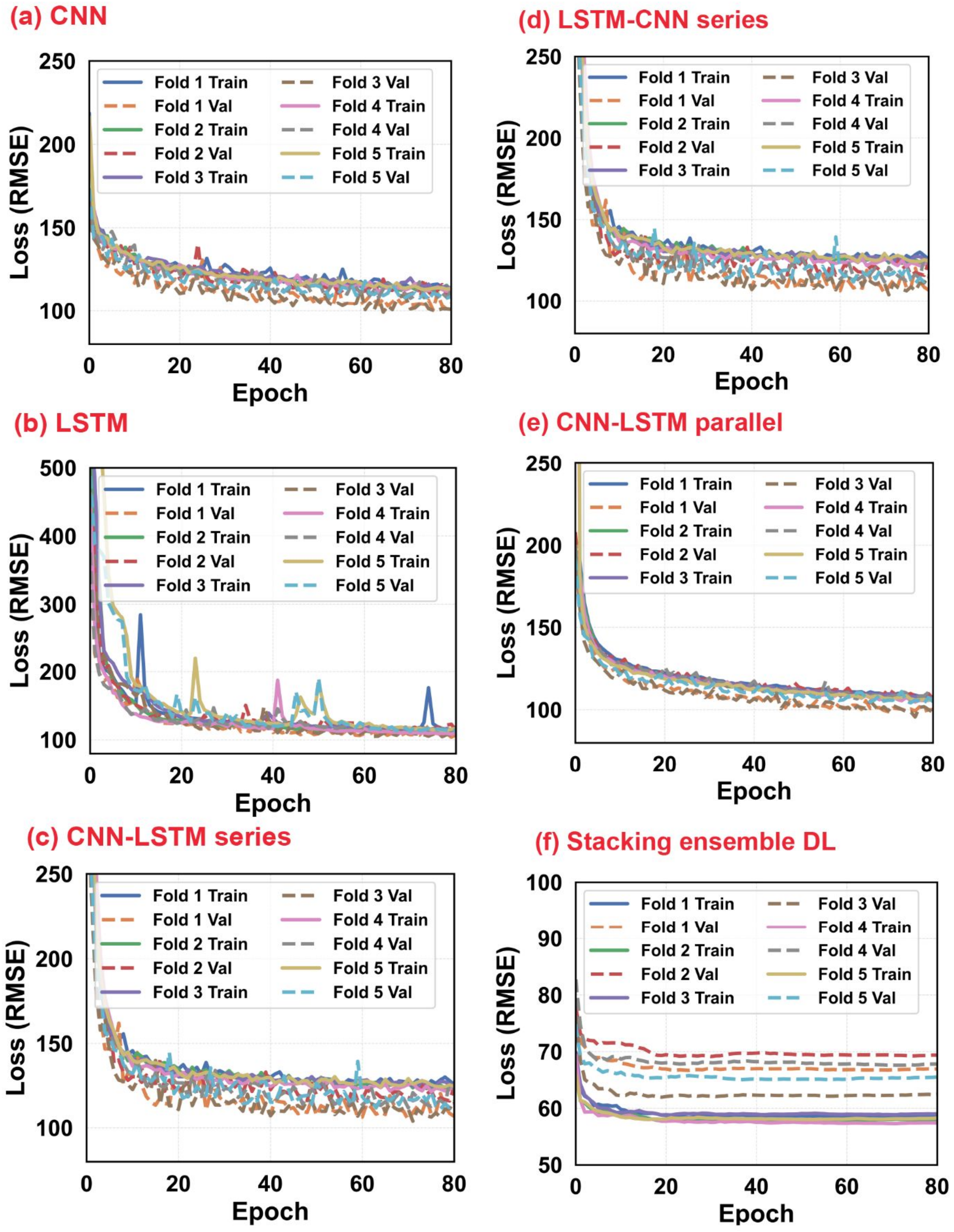

Figure S15: Training and validation loss comparison for all deep learning models across each of the 5 folds in cross-validation.

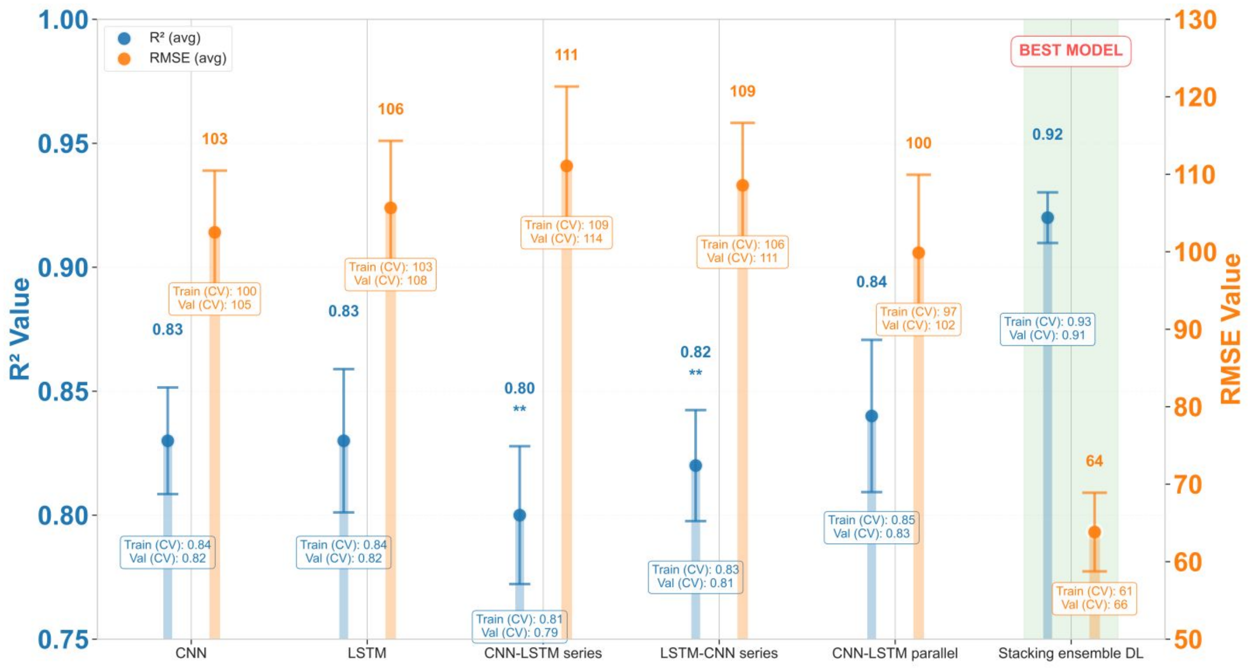

Figure S16: Predictive performance of various deep learning models using repeated five-fold cross-validation (five repeats). This approach evaluated model stability across different partitions of the dataset, yielding a mean R^2 of 0.91 and RMSE values comparable to the holdout validation, confirming the robustness and generalizability of the model within the compiled lab-scale dataset.

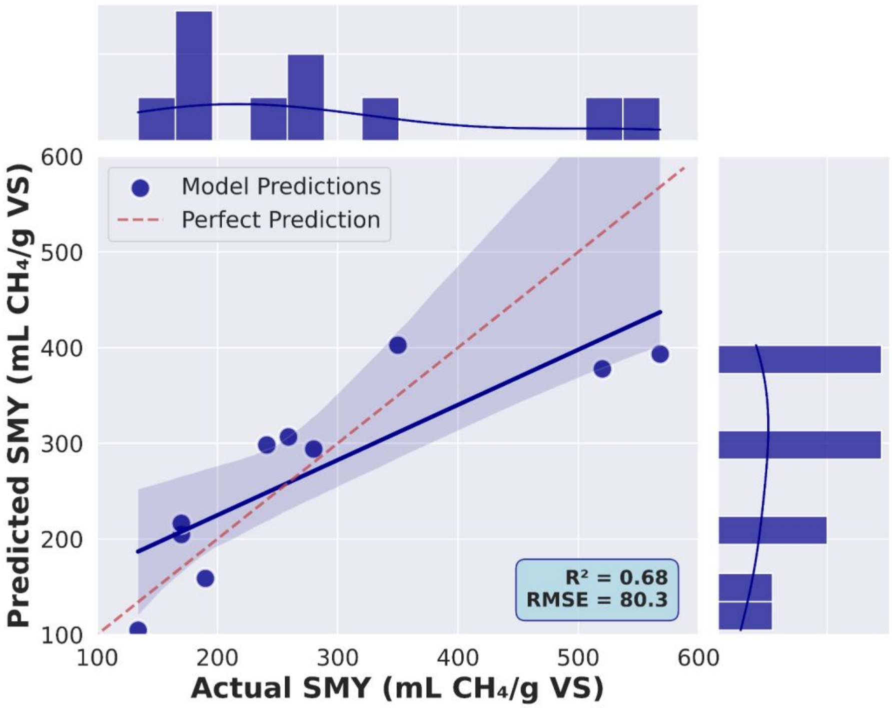

Figure S17: External validation of the stacking ensemble model on 10 biochar-augmented anaerobic digestion (AD) samples drawn from three independent, recent lab-scale studies not used during model training or internal validation. These studies represented diverse biochar types, dosages, and AD operational conditions. The model achieved a mean R^2 of 0.68 and RMSE of 80.3 mL CH_4/g VS, indicating good prediction accuracy and its generalizability across varied, previously unseen biochar-augmented lab-scale AD systems.

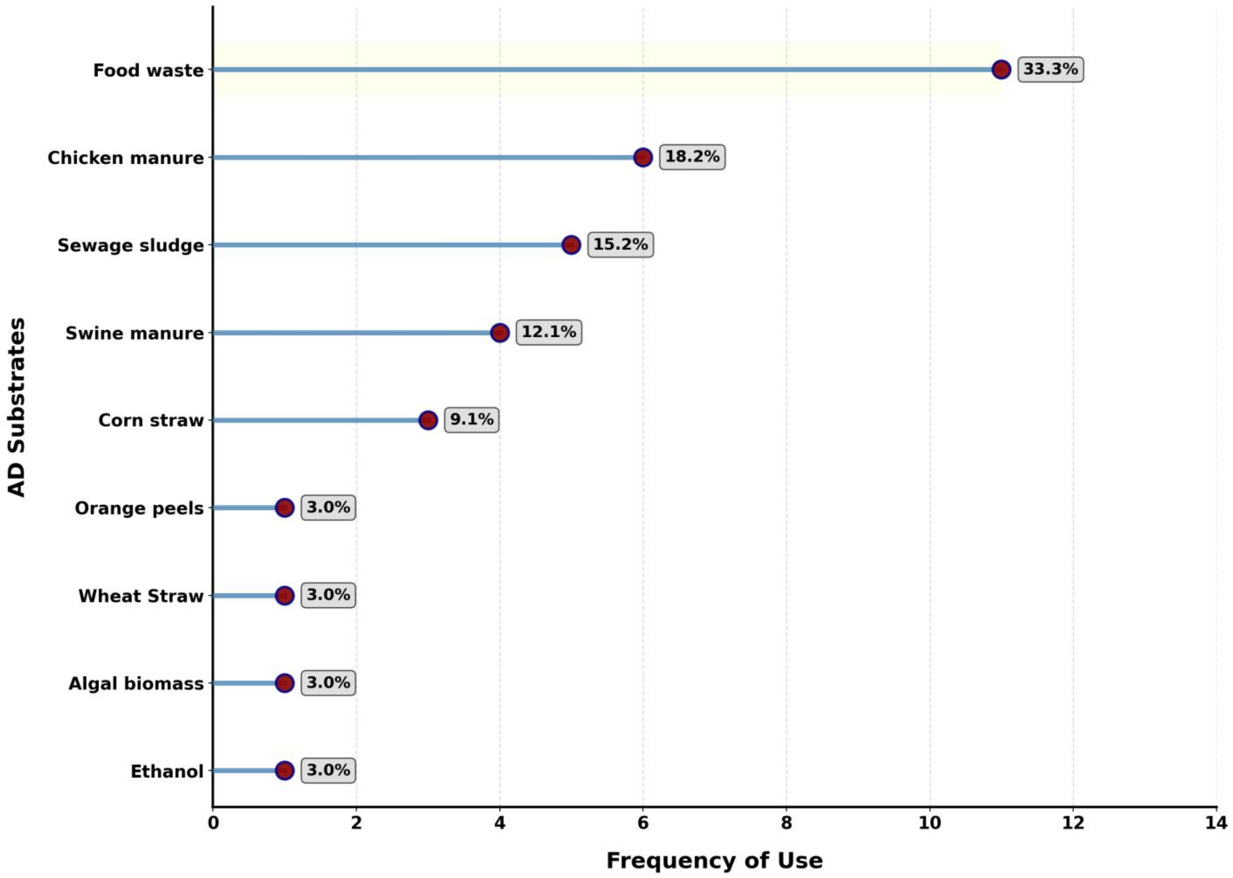

Figure S18: Frequency distribution of anaerobic digestion substrates used across studies. The plot shows the relative usage of different substrate types, where food waste includes kitchen waste and food processing residues (33.3%); chicken manure encompasses both fresh and raw chicken manure (18.2%); sewage sludge combines primary sludge, mixed sludge, and municipal sewage sludge (15.2%); swine manure represents all pig-related wastes including pig manure, swine manure, and swine waste (12.1%); and corn straw includes corn stover, cornstalk, and corn straw residues (9.1%). Other substrates, including orange peels, wheat straw, algal biomass, and ethanol, each represent 3.0% of the studies.

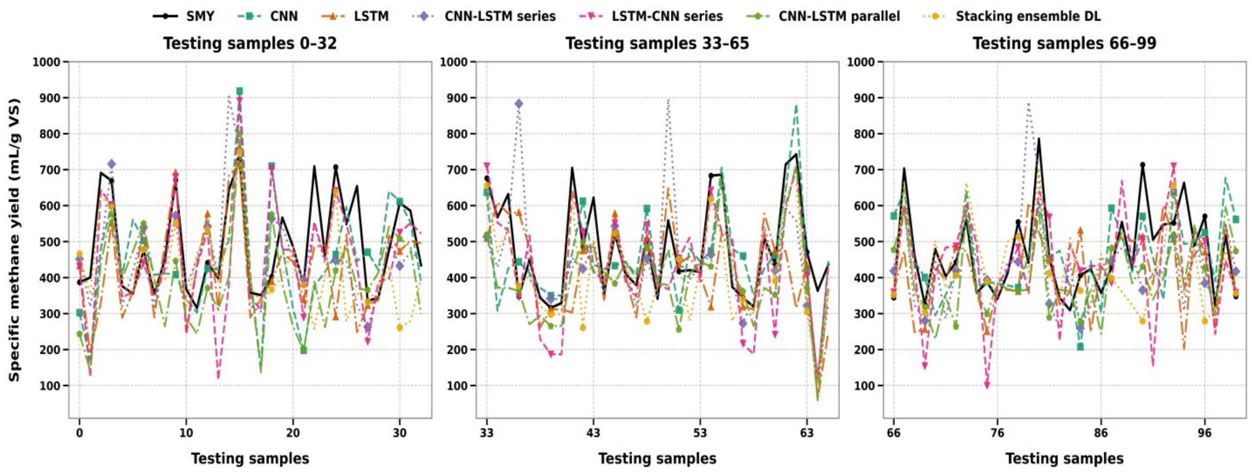

Figure S19: Variations of specific methane yield (SMY) predicted by stacking ensemble DL model over 100 experimental data points

Table S1: Sources of biochar-augmented anaerobic digestion dataset

No.	Title	DOI
1	Co-digestion of sludge and citrus peel wastes: Evaluating the effect of biochar addition on microbial communities.	https://doi.org/10.1016/j.bej.2018.06.010
2	Biochar enhanced thermophilic anaerobic digestion of food waste: Focusing on biochar particle size, microbial community analysis and pilot-scale application.	https://doi.org/10.1016/j.enconman.2020.112654
3	Enhanced methane production and syntrophic connection between microorganisms during semi-continuous anaerobic digestion of chicken manure by adding biochar.	https://doi.org/10.1016/j.enconman.2020.112654
4	Combined effects of liquid digestate recirculation and biochar on methane yield, enzyme activity, and microbial community during semi-continuous anaerobic digestion.	https://doi.org/10.1016/j.biortech.2022.128042
5	Enhancing anaerobic digestion of kitchen wastes with biochar: Link between different properties and critical mechanisms of promoting interspecies electron transfer.	https://doi.org/10.1016/j.renene.2020.11.153
6	Biochar triggering multipath methanogenesis and subdued propionic acid accumulation during semi-continuous anaerobic digestion.	https://doi.org/10.1016/j.biortech.2019.122026
7	Redox-active biochar facilitates potential electron transfer between syntrophic partners to enhance anaerobic digestion under high organic loading rate.	https://doi.org/10.1016/j.biortech.2019.122524
8	Methane yield enhancement of mesophilic and thermophilic anaerobic co-digestion of algal biomass and food waste using algal biochar: Semi-continuous operation and microbial community analysis.	https://doi.org/10.1016/j.biortech.2020.122892
9	Enhanced high-quality biomethane production from anaerobic digestion of primary sludge by corn stover biochar.	https://doi.org/10.1016/j.biortech.2020.123159
10	Methanogenic pathway and microbial succession during start-up and stabilization of thermophilic food waste anaerobic digestion with biochar.	https://doi.org/10.1016/j.biortech.2020.123751
11	Simultaneous carbon dioxide reduction and enhancement of methane production in biogas via anaerobic digestion of cornstalk in continuous stirred-tank reactors: The influences of biochar, environmental parameters, and microorganisms.	https://doi.org/10.1016/j.biortech.2020.124146
12	Anaerobic co-digestion of corn stover and chicken manure using continuous stirred tank reactor: The effect of biochar addition and urea pretreatment.	https://doi.org/10.1016/j.biortech.2020.124197

13	Semi-continuous anaerobic digestion of mixed wastewater sludge with biochar addition.	https://doi.org/10.1016/j.biortech.2021.125664
14	Effect of biochar on reactor performance and methane generation during the anaerobic digestion of food waste treatment at long-run operations.	https://doi.org/10.1016/j.jece.2019.103067
15	Internal enhancement mechanism of biochar with graphene structure in anaerobic digestion: The bioavailability of trace elements and potential direct interspecies electron transfer.	https://doi.org/10.1016/j.jece.2019.103067
16	Internal enhancement mechanism of biochar with graphene structure in anaerobic digestion: The bioavailability of trace elements and potential direct interspecies electron transfer.	https://doi.org/10.1016/j.cej.2020.126833
17	Sawdust-derived biochar much mitigates VFAs accumulation and improves microbial activities to enhance methane production in thermophilic anaerobic digestion.	https://doi.org/10.1021/acssuschemeng.8b04789
18	Effect of organic loading rate on the anaerobic digestion of swine waste with biochar addition.	https://doi.org/10.1007/s11356-021-13428-1
19	Pyrolyzed waste stream and biochar performance evaluation in food waste anaerobic digestion.	https://doi.org/10.1007/s10098-020-01862-7
20	Monitoring of food waste anaerobic digestion performance: Conventional co-substrates vs. unmarketable biochar additions.	https://doi.org/10.3390/foods10102353
21	Jointly reducing antibiotic resistance genes and improving methane yield in anaerobic digestion of chicken manure by feedstock microwave pretreatment and activated carbon supplementation.	https://doi.org/10.1016/j.cej.2019.04.207
22	Bioaugmentation of *Methanosarcina thermophila* grown on biochar particles during semi-continuous thermophilic food waste anaerobic digestion under two different bioaugmentation regimes.	https://doi.org/10.1016/j.biortech.2022.127590
23	Syntrophic consortium with the aid of coconut shell-derived biochar enhances methane recovery from ammonia-inhibited anaerobic digestion.	https://doi.org/10.1016/j.scitotenv.2023.162182
24	A novel strategy to reduce trace element supplementation in the semi-solid anaerobic digestion with gradient ammonia concentration: the role of biochar.	https://doi.org/10.1016/j.fuel.2022.127332
25	Evaluation of digestate-derived biochar to alleviate ammonia inhibition during long-term anaerobic digestion of food waste.	https://doi.org/10.1016/j.chemosphere.2022.137150
26	Enhancement effect of biochar addition on anaerobic co-digestion of pig manure and corn straw under biogas slurry circulation.	https://doi.org/10.1016/j.biortech.2023.128654

External validation	Sustained methane production enhancement by magnetic biochar and its recovery in semi-continuous anaerobic digestion with varying substrate C/N ratios	https://doi.org/10.1016/j.cej.2025.163050
External validation	Rethinking the biochar impact on the anaerobic digestion of food waste in bench-scale digester: Spatial distribution and biogas production	https://doi.org/10.1016/j.biortech.2025.132115
External validation	Overcoming ammonia inhibition via biochar-assisted anaerobic co-digestion of thermally-treated thickened waste activated sludge and food waste	https://doi.org/10.1016/j.jenvman.2024.123909

Table S2: Raw dataset ranges collected from experimental studies

	Parameters	Abbreviation	Units	Ranges
Anaerobic digestion parameters	Organic loading rate	OLR	gVS/L.d	0.60 – 20
	Hydraulic retention time	HRT	Day	2 – 80
	Temperature	T	^{o}C	35 – 55
	Carbon/nitrogen	C/N	–	6.54 – 35.35
	Total solids	TS	%	2.13 – 92.48
	Volatile solids	VS	%	1.38 – 94
	AD substrate particle size	PS_AD	mm	1 – 5
Biochar parameters	Biochar dosage	BD	g/L	0.01 – 50
	Specific surface area	SSA	m^2/g	2.60 – 511
	Pore volume	PV	cm^3/g	0.01 – 0.22
	Biochar particle size	PS_B	–	0.05 – 4
	Biochar pH	pH_B	–	7 – 10.95
	Electrical conductivity	EC	mS/m	0.01 – 280
	Moisture content	MC	%	1.59 – 36
	Volatile matter	VM	%	6 – 94.88
	Fixed carbon	FC	%	2.36 – 79.44
	Ash	Ash	%	0.30 – 84
	Carbon	C	%	5.56 – 82.10
	Hydrogen	H	%	0.10 – 8.42
	Nitrogen	N	%	0.10 – 7.89
	Oxygen	O	%	0.06 – 44.73
Target parameter	Specific methane yield	SMY	mL CH_4/g VS	20 – 1375

Table S3: Optimized hyperparameters

**Models**	**Optimized hyperparamters**
CNN	{'filters': 73, 'kernel_size': 6, 'units': 73, 'dropout_rate': .136, 'learning_rate': 0.0758, 'epochs': 70, 'batch_size': 49}
LSTM	{'units': 66, 'dense_units': 83, 'dropout_rate': 0.255, 'learning_rate': 0.0068, 'epochs': 82, 'batch_size': 33}
CNN-LSTM series	{'filters': 112, 'kernel_size': 5, 'units': 127, 'dense_units': 184, 'dropout_rate': 0.282, 'learning_rate': 0.00059, 'epochs': 41, 'batch_size': 64}
LSTM-CNN series	{'units': 128, 'filters': 63, 'kernel_size': 4, 'dense_units': 191, 'dropout_rate': 0.378, 'learning_rate': 0.00093, 'epochs': 33, 'batch_size': 19}
CNN-LSTM parallel	{'filters': 113, 'kernel_size': 4, 'lstm_units': 43, 'units': 113, 'dropout_rate': 0.135, 'learning_rate': 0.00797, 'epochs': 95, 'batch_size': 26}
ExtraTreeRegressor	{'n_estimators': 85, 'max_depth': 30, 'min_samples_split': 5, 'min_samples_leaf': 1, 'max_features': None}

Table S4: Summary of external validation dataset

Parameter	count	mean	std	min	25%	50%	75%	max
OLR	10	3.9	2.28	2	2.25	3	4	8
HRT	10	21.2	2.53	20	20	20	20	26
T	10	36.6	0.84	35	37	37	37	37
C/N	10	18.43	2.54	13.77	18.4	20	20	20
TS	10	12.55	6.37	9	9	9.9	10.25	24.59
VS	10	10.42	4.659	7.5	8.1	8.1	9.2	19.19
PS_AD	10	1.77	0.485	0.85	2	2	2	2
BD	10	5.64	6.143	0.8	0.8	1.6	10	15
SA	10	242.04	133.31	41.98	142.2	342	342	342
PV	10	0.172	0.047	0.093	0.15	0.205	0.205	0.205
PS_B	10	0.395	0.375	0.125	0.25	0.25	0.25	1.1
pH_B	10	10.288	0.36	9.62	10.32	10.5	10.5	10.5
EC	10	2.25	0.667	1.75	2	2	2	3.5
MC	10	3.726	1.083	3	3	3	4	5.63
VM	10	18.702	2.534	17.5	17.5	17.5	17.5	23.51
FC	10	58.05	17.25	32.25	45	71	71	71
Ash	10	17.622	13.20	7.5	7.5	7.5	30	35.61
C	10	62.96	7.03	49.68	65	66.7	66.7	66.7
H	10	1.502	0.163	1.26	1.5	1.5	1.5	1.75
N	10	1.90	2.34	0.5	0.5	0.5	1.75	6.23
O	10	29.22	0.944	28.5	28.5	28.5	30	30.58
SMY	10	288.2	149.20	134	175	250	332.5	568

Table S5: Summary of machine learning-based studies on biochar-augmented anaerobic digestion (AD) systems

References	Operation mode (Batch/semi-continuous)	AD feedstock	Data size	ML algorithm	Input parameters	Output parameters	Results (Best model)
7	Batch	Single feedstock (Sewage sludge)	51	ANN	Biochar source, SA, TPV, APV, APD, C, H, N, O, Ash, BD, AD substrate TS, VS, I:S, T	Cumulative methane production	R^2 ~ 0.99
8	Batch	2 feedstock types (Cheese whey and septage)	25	ANN	TS and BD	Cumulative methane yield	R^2 ~ 0.99 MSE ~ 18
9	Batch	2 feedstock types (Poultry litter and wheat straw)	17	ANN, RSM, GA	C/N, TS, and BD	Methane yield	RSM (R^2 ~ 0.99, RMSE ~ 0.91
10	Batch	2 feedstock types (Cattle manure and green algae)	14	ANN, RSM	DT and BD	Biogas yield	ANN (R^2 ~ 0.99, RMSE~0.55)
11	Batch	Multiple feedstocks (e.g., sewage sludge and animal manure)	228	XGBoost, RF, GBR	Biochar SSA, PS, pH_B, EC, C, H, O, N, ash, BD, AD substrate TS, VS, SM, T, pH_AD, DT	Methane yield and Rmax	R^2 ~ 0.69-0.84
12	Batch	4 feedstock types (Beer lees, Manure, Food waste, Waste sludge)	123	TPOT, H2O, GLM, GBM, RF, ANN	PS_AD, EC, BC, BD, FSC, T, DT	Methane yield	GBR (R^2 ~ 0.96)
13	Semi-continuous	4 feedstock types (Food wastes, manure, algal residue, corn stover)	715	KNN, RF, GBR, SVR, ANN, ensemble	C/N, VS/TS, OLR, HRT, T, BD, broth pH, COD	Methane yield and content	Ensemble model (R^2 ~ 0.82-0.86)
**This study**	Semi-continuous	15 feedstock types (including food waste, sewage sludge, manure, agricultural residues, etc.)	~5000	CNN, LSTM, CNN-LSTM-series, LSTM-CNN-series, CNN-LSTM-parallel, and SE	AD substrate TS, VS, C/N, OLR, HRT, T, PS_AD, EC, SSA, PV, pH_B, PS_B, VM, FC, MC, ash, C, H, N, O	Specific methane yield	SE model (R^2 ~ 0.94 RMSE ~ 62)

Note: SA: Surface area; TPV: Total pore volume; APV: Average pore volume; APD: Average pore diameter; C: Carbon; H: Hydrogen; N: Nitrogen; O: Oxygen; C/N: Carbon-to-nitrogen ratio; BD: Biochar dosage; TS: Total solids; VS: Volatile solids; FC: Fixed carbon; I:S: Inoculum to substrate ratio; T: Digester temperature; SM: Substrate mass; pH_AD: Digester pH; DT: Digestion time; PS_AD: Substrate particle size; EC: Electrical conductivity; BC: Biochar capacitance; FSC: Feedstock concentration; OLR: Organic loading rate; HRT: Hydraulic retention time; SSA: Specific surface area; PV: Pore volume; pH_B: Biochar pH; VM: Volatile matter; FC: Fixed carbon; MC: Moisture content; Rmax: Maximum methane production rate; ML: Machine learning; TPOT: Python automated machine learning tool; GLM: Generalized linear model; GBM: Gradient boosting machine; XGBoost: Extreme gradient boosting; RF: Random forests; ANN: Artificial neural network; SE: Stacking ensemble; CNN: Convolutional neural network; LSTM: Long short-term memory; KNN: K-nearest neighbors; SVR: Support vector regression; RSM: Response surface methodology

References

(1) Zhang, Y.; Feng, Y.; Ren, Z.; Zuo, R.; Zhang, T.; Li, Y.; Wang, Y.; Liu, Z.; Sun, Z.; Han, Y.; Feng, L.; Aghbashlo, M.; Tabatabaei, M.; Pan, J. Tree-Based Machine Learning Model for Visualizing Complex Relationships between Biochar Properties and Anaerobic Digestion. *Bioresour. Technol.* **2023**, *374* (February), 128746. https://doi.org/10.1016/j.biortech.2023.128746.

(2) Deng, Y.; Zhang, Y.; Zhao, Z. A Data-Driven Approach for Revealing the Linkages between Differences in Electrochemical Properties of Biochar during Anaerobic Digestion Using Automated Machine Learning. *Sci. Total Environ.* **2024**, *927* (February), 172291. https://doi.org/10.1016/j.scitotenv.2024.172291.

(3) Hong, S.; Lynn, H. S. Accuracy of Random-Forest-Based Imputation of Missing Data in the Presence of Non-Normality, Non-Linearity, and Interaction. *BMC Med. Res. Methodol.* **2020**, *20* (1), 1–12. https://doi.org/10.1186/s12874-020-01080-1.

(4) Li, J.; Zhang, L.; Li, C.; Tian, H.; Ning, J.; Zhang, J.; Tong, Y. W.; Wang, X. Data-Driven Based In-Depth Interpretation and Inverse Design of Anaerobic Digestion for CH4-Rich Biogas Production. *ACS ES T Eng.* **2022**, *2* (4), 642–652. https://doi.org/10.1021/acsestengg.1c00316.

(5) Fagbohungbe, M. O.; Herbert, B. M. J.; Hurst, L.; Ibeto, C. N.; Li, H.; Usmani, S. Q.; Semple, K. T. The Challenges of Anaerobic Digestion and the Role of Biochar in Optimizing Anaerobic Digestion. *Waste Manag.* **2017**, *61*, 236–249. https://doi.org/10.1016/j.wasman.2016.11.028.

(6) Zhang, P.; Zheng, S.; Liu, J.; Wang, B.; Liu, F.; Feng, Y. Surface Properties of Activated Sludge-Derived Biochar Determine the Facilitating Effects on Geobacter Co-Cultures. *Water Res.* **2018**, *142*, 441–451. https://doi.org/10.1016/j.watres.2018.05.058.

(7) Tag, A. T.; Duman, G.; Ucar, S.; Yanik, J. Effects of Feedstock Type and Pyrolysis Temperature on Potential Applications of Biochar. *J. Anal. Appl. Pyrolysis* **2016**, *120*, 200–206. https://doi.org/10.1016/j.jaap.2016.05.006.

(8) Li, Q.; Zhang, X.; Mao, M.; Wang, X.; Shang, J. Carbon Content Determines the Aggregation of Biochar Colloids from Various Feedstocks. *Sci. Total Environ.* **2023**, *880* (March), 163313. https://doi.org/10.1016/j.scitotenv.2023.163313.

(9) Masebinu, S. O.; Akinlabi, E. T.; Muzenda, E.; Aboyade, A. O. A Review of Biochar Properties and Their Roles in Mitigating Challenges with Anaerobic Digestion. *Renew. Sustain. Energy Rev.* **2019**, *103* (September 2018), 291–307. https://doi.org/10.1016/j.rser.2018.12.048.

(10) Yi, J.; Dong, B.; Jin, J.; Dai, X. Effect of Increasing Total Solids Contents on Anaerobic Digestion of Food Waste under Mesophilic Conditions: Performance and Microbial Characteristics Analysis. *PLoS One* **2014**, *9* (7). https://doi.org/10.1371/journal.pone.0102548.

(11) Zhao, W.; Yang, H.; He, S.; Zhao, Q.; Wei, L. A Review of Biochar in Anaerobic Digestion to Improve Biogas Production: Performances, Mechanisms and Economic Assessments. *Bioresour. Technol.* **2021**, *341* (August), 125797. https://doi.org/10.1016/j.biortech.2021.125797.

(12) Kumar, M.; Dutta, S.; You, S.; Luo, G.; Zhang, S.; Show, P. L.; Sawarkar, A. D.; Singh, L.; Tsang, D. C. W. A Critical Review on Biochar for Enhancing Biogas Production from Anaerobic Digestion of Food Waste and Sludge. *J. Clean. Prod.* **2021**, *305*, 127143. https://doi.org/10.1016/j.jclepro.2021.127143.

(13) Jia, R.; Song, Y. C.; Piao, D. M.; Kim, K.; Lee, C. Y.; Park, J. Exploration of Deep Learning

Models for Real-Time Monitoring of State and Performance of Anaerobic Digestion with Online Sensors. *Bioresour. Technol.* **2022**, *363* (August), 127908. https://doi.org/10.1016/j.biortech.2022.127908.

(14) Jeong, K.; Abbas, A.; Shin, J.; Son, M.; Kim, Y. M.; Cho, K. H. Prediction of Biogas Production in Anaerobic Co-Digestion of Organic Wastes Using Deep Learning Models. *Water Res.* **2021**, *205* (September), 117697. https://doi.org/10.1016/j.watres.2021.117697.

(15) Khan, M.; Ahmad, I.; Ahsan, M.; Kano, M.; Caliskan, H. Prediction of Optimum Operating Conditions of a Furnace under Uncertainty: An Integrated Framework of Artificial Neural Network and Genetic Algorithm. *Fuel* **2022**, *330* (August), 125563. https://doi.org/10.1016/j.fuel.2022.125563.

(16) Khan, M.; Ullah, Z.; Mašek, O.; Raza Naqvi, S.; Nouman Aslam Khan, M. Artificial Neural Networks for the Prediction of Biochar Yield: A Comparative Study of Metaheuristic Algorithms. *Bioresour. Technol.* **2022**, *355* (February). https://doi.org/10.1016/j.biortech.2022.127215.

(17) Altmann, A.; Toloşi, L.; Sander, O.; Lengauer, T. Permutation Importance: A Corrected Feature Importance Measure. *Bioinformatics* **2010**, *26* (10), 1340–1347. https://doi.org/10.1093/bioinformatics/btq134.

(18) Khan, M.; Ullah, Z.; Mašek, O.; Raza Naqvi, S.; Nouman Aslam Khan, M. Artificial Neural Networks for the Prediction of Biochar Yield: A Comparative Study of Metaheuristic Algorithms. *Bioresour. Technol.* **2022**, *355* (April). https://doi.org/10.1016/j.biortech.2022.127215.

(19) Ekanayake, I. U.; Meddage, D. P. P.; Rathnayake, U. A Novel Approach to Explain the Black-Box Nature of Machine Learning in Compressive Strength Predictions of Concrete Using Shapley Additive Explanations (SHAP). *Case Stud. Constr. Mater.* **2022**, *16* (April), e01059. https://doi.org/10.1016/j.cscm.2022.e01059.

(20) Priyadarshi, R.; Ranjan, R. *Evolution of Swarm Intelligence : A Systematic Review of Particle Swarm and Ant Colony Optimization Approaches in Modern Research*; Springer Netherlands, 2025. https://doi.org/10.1007/s11831-025-10247-2.

(21) Wang, H.; Yang, S.; Ip, W. H.; Wang, D. A Particle Swarm Optimization Based Memetic Algorithm for Dynamic Optimization Problems. *Nat. Comput.* **2010**, *9* (3), 703–725. https://doi.org/10.1007/s11047-009-9176-2.

(22) Khashaba, N. H.; Ettouney, R. S.; Abdelaal, M. M.; Ashour, F. H.; El-Rifai, M. A. Artificial Neural Network Modeling of Biochar Enhanced Anaerobic Sewage Sludge Digestion. *J. Environ. Chem. Eng.* **2022**, *10* (4), 107988. https://doi.org/10.1016/j.jece.2022.107988.

(23) K, B.; Pilli, S.; Rao, P. V.; Tyagi, R. D. Predictive Modelling of Methane Yield in Biochar-Amended Cheese Whey and Septage Co-Digestion: Exploring Synergistic Effects Using Gompertz and Neural Networks. *Chemosphere* **2024**, *353* (February), 141558. https://doi.org/10.1016/j.chemosphere.2024.141558.

(24) Zhan, Y.; Zhu, J. Response Surface Methodology and Artificial Neural Network-Genetic Algorithm for Modeling and Optimization of Bioenergy Production from Biochar-Improved Anaerobic Digestion. *Appl. Energy* **2024**, *355* (July 2023), 122336. https://doi.org/10.1016/j.apenergy.2023.122336.

(25) Şenol, H.; Çolak, E.; Elibol, E. A.; Hassaan, M. A.; El Nemr, A. Optimisation of Biochar Dose in Anaerobic Co-Digestion of Green Algae and Cattle Manure Using Artificial Neural Networks and Response Surface Methodology. *Chem. Eng. J.* **2024**, *493* (May).

https://doi.org/10.1016/j.cej.2024.152750.

References (Sources of dataset collected in this study)

1) Martínez, E. J., Rosas, J. G., Sotres, A., Moran, A., Cara, J., Sánchez, M. E., & Gómez, X. (2018). Codigestion of sludge and citrus peel wastes: Evaluating the effect of biochar addition on microbial communities. Biochemical Engineering Journal, 137, 314-325. https://doi.org/10.1016/j.bej.2018.06.010
2) Zhang, L., Lim, E. Y., Loh, K. C., Ok, Y. S., Lee, J. T., Shen, Y., ... & Tong, Y. W. (2020). Biochar enhanced thermophilic anaerobic digestion of food waste: Focusing on biochar particle size, microbial community analysis and pilot-scale application. Energy Conversion and Management, 209, 112654. https://doi.org/10.1016/j.enconman.2020.112654
3) Pan, J., Ma, J., Zhai, L., & Liu, H. (2019). Enhanced methane production and syntrophic connection between microorganisms during semi-continuous anaerobic digestion of chicken manure by adding biochar. Journal of Cleaner Production, 240, 118178. https://doi.org/10.1016/j.jclepro.2019.118178
4) Shao, Z., Chen, H., Zhao, Z., Yang, Z., Qiu, L., & Guo, X. (2022). Combined effects of liquid digestate recirculation and biochar on methane yield, enzyme activity, and microbial community during semi-continuous anaerobic digestion. Bioresource Technology, 364, 128042. https://doi.org/10.1016/j.biortech.2022.128042
5) Wang, J., Zhao, Z., & Zhang, Y. (2021). Enhancing anaerobic digestion of kitchen wastes with biochar: Link between different properties and critical mechanisms of promoting interspecies electron transfer. Renewable Energy, 167, 791-799. https://doi.org/10.1016/j.renene.2020.11.153
6) Ma, J., Pan, J., Qiu, L., Wang, Q., & Zhang, Z. (2019). Biochar triggering multipath methanogenesis and subdued propionic acid accumulation during semi-continuous anaerobic digestion. Bioresource technology, 293, 122026. https://doi.org/10.1016/j.biortech.2019.122026
7) Wang, G., Li, Q., Li, Y., Xing, Y., Yao, G., Liu, Y., ... & Wang, X. C. (2020). Redox-active biochar facilitates potential electron tranfer between syntrophic partners to enhance anaerobic digestion under high organic loading rate. Bioresource Technology, 298, 122524. https://doi.org/10.1016/j.biortech.2019.122524
8) Zhang, L., Li, F., Kuroki, A., Loh, K. C., Wang, C. H., Dai, Y., & Tong, Y. W. (2020). Methane yield enhancement of mesophilic and thermophilic anaerobic co-digestion of algal biomass and food waste using algal biochar: Semi-continuous operation and microbial community analysis. Bioresource technology, 302, 122892. https://doi.org/10.1016/j.biortech.2020.122892
9) Wei, W., Guo, W., Ngo, H. H., Mannina, G., Wang, D., Chen, X., ... & Ni, B. J. (2020). Enhanced high-quality biomethane production from anaerobic digestion of primary sludge by corn stover biochar. Bioresource technology, 306, 123159. https://doi.org/10.1016/j.biortech.2020.123159
10) Lim, E. Y., Tian, H., Chen, Y., Ni, K., Zhang, J., & Tong, Y. W. (2020). Methanogenic pathway and microbial succession during start-up and stabilization of thermophilic food waste anaerobic digestion with biochar. Bioresource Technology, 314, 123751. https://doi.org/10.1016/j.biortech.2020.123751
11) Shen, R., Jing, Y., Feng, J., Zhao, L., Yao, Z., Yu, J., ... & Chen, R. (2021). Simultaneous carbon dioxide reduction and enhancement of methane production in biogas via anaerobic digestion of cornstalk in continuous stirred-tank reactors: The influences of biochar, environmental parameters, and microorganisms. Bioresource technology, 319, 124146. https://doi.org/10.1016/j.biortech.2020.124146

12) Yu, Q., Sun, C., Liu, R., Yellezuome, D., Zhu, X., Bai, R., ... & Sun, M. (2021). Anaerobic co-digestion of corn stover and chicken manure using continuous stirred tank reactor: The effect of biochar addition and urea pretreatment. Bioresource Technology, 319, 124197. https://doi.org/10.1016/j.biortech.2020.124197
13) Chiappero, M., Berruti, F., Mašek, O., & Fiore, S. (2021). Semi-continuous anaerobic digestion of mixed wastewater sludge with biochar addition. Bioresource technology, 340, 125664. https://doi.org/10.1016/j.biortech.2021.125664
14) Xu, S., Duan, Y., Zou, S., Liu, H., Luo, L., & Wong, J. W. (2022). Evaluations of biochar amendment on anaerobic co-digestion of pig manure and sewage sludge: waste-to-methane conversion, microbial community, and antibiotic resistance genes. Bioresource Technology, 346, 126400. https://doi.org/10.1016/j.biortech.2021.126400
15) Giwa, A. S., Xu, H., Chang, F., Wu, J., Li, Y., Ali, N., ... & Wang, K. (2019). Effect of biochar on reactor performance and methane generation during the anaerobic digestion of food waste treatment at long-run operations. Journal of Environmental Chemical Engineering, 7(4), 103067. https://doi.org/10.1016/j.jece.2019.103067
16) Qi, Q., Sun, C., Zhang, J., He, Y., & Tong, Y. W. (2021). Internal enhancement mechanism of biochar with graphene structure in anaerobic digestion: The bioavailability of trace elements and potential direct interspecies electron transfer. Chemical Engineering Journal, 406, 126833. https://doi.org/10.1016/j.cej.2020.126833
17) Wang, G., Li, Q., Gao, X., & Wang, X. C. (2018). Sawdust-derived biochar much mitigates VFAs accumulation and improves microbial activities to enhance methane production in thermophilic anaerobic digestion. ACS Sustainable Chemistry & Engineering, 7(2), 2141-2150. https://doi.org/10.1021/acssuschemeng.8b04789
18) Sánchez, E., Herrmann, C., Maja, W., & Borja, R. (2021). Effect of organic loading rate on the anaerobic digestion of swine waste with biochar addition. Environmental science and pollution research, 28, 38455-38465. https://doi.org/10.1007/s11356-021-13428-1
19) Giwa, A. S., Zhang, X., Xu, H., Vakili, M., Yuan, J., & Wang, K. (2020). Pyrolyzed waste stream and biochar performance evaluation in food waste anaerobic digestion. Clean Technologies and Environmental Policy, 22, 1199-1211. https://doi.org/10.1007/s10098-020-01862-7
20) Chaher, N. E. H., Nassour, A., Hamdi, M., & Nelles, M. (2021). Monitoring of food waste anaerobic digestion performance: Conventional co-substrates vs. unmarketable biochar additions. Foods, 10(10), 2353. https://doi.org/10.3390/foods10102353
21) Zhang, L., Loh, K. C., & Zhang, J. (2019). Jointly reducing antibiotic resistance genes and improving methane yield in anaerobic digestion of chicken manure by feedstock microwave pretreatment and activated carbon supplementation. Chemical Engineering Journal, 372, 815-824. https://doi.org/10.1016/j.cej.2019.04.207
22) Lee, J. T., Dutta, N., Zhang, L., Tsui, T. T., Lim, S., Tio, Z. K., ... & Tong, Y. W. (2022). Bioaugmentation of Methanosarcina thermophila grown on biochar particles during semi-continuous thermophilic food waste anaerobic digestion under two different bioaugmentation regimes. Bioresource technology, 360, 127590. https://doi.org/10.1016/j.biortech.2022.127590
23) Cai, Y., Shen, X., Meng, X., Zheng, Z., Usman, M., Hu, K., & Zhao, X. (2023). Syntrophic consortium with the aid of coconut shell-derived biochar enhances methane recovery from ammonia-inhibited anaerobic digestion. Science of the Total Environment, 872, 162182. https://doi.org/10.1016/j.scitotenv.2023.162182
24) Cai, Y., Meng, X., Hu, K., Zhao, X., Usman, M., Esposito, G., ... & Chen, S. (2023). A novel strategy to reduce trace element supplementation in the semi-solid anaerobic digestion with gradient ammonia concentration: the role of biochar. Fuel, 338, 127332. https://doi.org/10.1016/j.fuel.2022.127332

25) Peng, Y., Li, L., Dong, Q., Yang, P., Liu, H., Ye, W., ... & Peng, X. (2023). Evaluation of digestate-derived biochar to alleviate ammonia inhibition during long-term anaerobic digestion of food waste. Chemosphere, 311, 137150. https://doi.org/10.1016/j.chemosphere.2022.137150

26) Huang, X., Miao, X., Chu, X., Luo, L., Zhang, H., & Sun, Y. (2023). Enhancement effect of biochar addition on anaerobic co-digestion of pig manure and corn straw under biogas slurry circulation. Bioresource Technology, 372, 128654. https://doi.org/10.1016/j.biortech.2023.128654
